# Diversity and Activity of Alternative Nitrogenases in Sequenced Genomes and Coastal Environments

**DOI:** 10.3389/fmicb.2017.00267

**Published:** 2017-02-28

**Authors:** Darcy L. McRose, Xinning Zhang, Anne M. L. Kraepiel, François M. M. Morel

**Affiliations:** ^1^Department of Geosciences, Princeton University, PrincetonNJ, USA; ^2^Department of Chemistry, Princeton University, PrincetonNJ, USA

**Keywords:** alternative nitrogenase, PacBio, nitrogen fixation, AnfD, VnfD, NifD, ISARA

## Abstract

The nitrogenase enzyme, which catalyzes the reduction of N_2_ gas to NH_4_^+^, occurs as three separate isozyme that use Mo, Fe-only, or V. The majority of global nitrogen fixation is attributed to the more efficient ‘canonical’ Mo-nitrogenase, whereas Fe-only and V-(‘alternative’) nitrogenases are often considered ‘backup’ enzymes, used when Mo is limiting. Yet, the environmental distribution and diversity of alternative nitrogenases remains largely unknown. We searched for alternative nitrogenase genes in sequenced genomes and used PacBio sequencing to explore the diversity of canonical (*nifD*) and alternative (*anfD* and *vnfD*) nitrogenase amplicons in two coastal environments: the Florida Everglades and Sippewissett Marsh (MA). Genome-based searches identified an additional 25 species and 10 genera not previously known to encode alternative nitrogenases. Alternative nitrogenase amplicons were found in both Sippewissett Marsh and the Florida Everglades and their activity was further confirmed using newly developed isotopic techniques. Conserved amino acid sequences corresponding to cofactor ligands were also analyzed in *anfD* and *vnfD* amplicons, offering insight into environmental variants of these motifs. This study increases the number of available *anfD* and *vnfD* sequences ∼20-fold and allows for the first comparisons of environmental Mo-, Fe-only, and V-nitrogenase diversity. Our results suggest that alternative nitrogenases are maintained across a range of organisms and environments and that they can make important contributions to nitrogenase diversity and nitrogen fixation.

## Introduction

Nitrogen fixation is a biogeochemically important process that shapes the fertility of marine and terrestrial environments. A need for adequate constraints on rates of nitrogen fixation across global ecosystems (Vitousek and Howarth, 1991; Gruber and Sarmiento, 1997; Cleveland et al., 1999; Codispoti et al., 2001) has spurred investigations into new locations for nitrogen fixation as well as novel nitrogen fixers. Biological nitrogen fixation is catalyzed by the nitrogenase enzyme, which can occur as three different isozymes, that use either Mo, Fe-only, or V at the active site (Bishop et al., 1986; Robson et al., 1986). While all known diazotrophs encode a canonical Mo-nitrogenase, some also encode an additional Fe-only nitrogenase, V-nitrogenase, or both. Due to their lower efficiency (Eady and Robson, 1984) compared with canonical nitrogenases, these ‘alternative’ nitrogenases are typically viewed as ‘backup’ enzymes, used only when Mo is not available. However, the exact conditions under which these enzymes are used in the environment remain unknown.

The structural components of the nitrogenase enzyme are encoded by *nifHDK* (Mo-nitrogenases), *anfHDK* (Fe-only nitrogenases), and *vnfHDK* (V-nitrogenase). Alternative nitrogenases also require *anf/vnfG*, for which there is no equivalent in Mo-nitrogenases (Waugh et al., 1995). The environmental diversity of Mo-nitrogenases has been studied extensively using polymerase chain reaction (PCR) primers targeting *nifH*, the standard functional gene for nitrogenase studies (Zehr et al., 2003; Gaby and Buckley, 2011). *NifH* studies occasionally detect sequences thought to belong to alternative nitrogenases (*anfH, vnfH*) (Zehr et al., 1995; Affourtit et al., 2001; Jenkins et al., 2004; Steward et al., 2004; Man-Aharonovich et al., 2007; Farnelid et al., 2009, 2013). Unfortunately, *nif/anf/vnfH* genes do not encode the region of the enzyme that harbors the metal-center and as such are not conclusively diagnostic for the type of isozyme (Young, 2005). In contrast, *nif/anf/vnfD* and *nif/anf/vnfK* can be used to distinguish between isozymes (Raymond et al., 2004; Young, 2005). However, very few studies (Loveless et al., 1999; Betancourt et al., 2008; Tan et al., 2009) have surveyed these genes in the environment, leaving alternative nitrogenase diversity largely unexplored.

Nonetheless, numerous lines of evidence suggest that alternative nitrogenases are active in the environment. Genes encoding alternative nitrogenases can be found in sequenced genomes from taxonomically diverse diazotrophs (Zehr et al., 2003; Raymond et al., 2004; Young, 2005; Dos Santos et al., 2012). Many of these organisms are in culture, and the functionality of their alternative nitrogenases has been demonstrated (Bishop et al., 1986; Thiel, 1993; Oda et al., 2005, 2008). In addition, organisms with alternative nitrogenases have been isolated from wood chips, soils, and mangrove sediments (Loveless et al., 1999; Betancourt et al., 2008). Expression of alternative nitrogenase genes has also been shown in the termite hindgut (*anfH*, Noda et al., 1999), lichen cyanobionts (*vnfDGN*, Hodkinson et al., 2014) and mesocosm soil experiments amended with vanadium (*vnfD*, Bellenger et al., 2014). Using a newly developed technique, the Isotopic Acetylene Reduction Assay (ISARA), which distinguishes between canonical and alternative N_2_-fixation by measuring ^13^C isotopes in the acetylene reduction assay, we recently reported the activity of alternative nitrogenases in Sippewissett Marsh (Zhang et al., 2016).

Here, we searched for alternative nitrogenases in sequenced genomes and used targeted single-molecule real-time sequencing (PacBio) of *nifD, anfD*, and *vnfD* amplicons to explore isozyme diversity in the environment. Careful investigations into the environmental origins of strains used in sequenced genome studies suggested that organisms with alternative nitrogenases have been isolated from a variety of locations. Our own sequencing efforts showed that alternative nitrogenase amplicons were present in samples from both the Florida Everglades and Sippewissett Marsh. Total nitrogenase activity and alternative isozyme contributions in environmental samples were verified with the traditional acetylene reduction assay (Dilworth, 1966) as well as the ISARA technique (Zhang et al., 2016). In order to understand possible trace metal drivers of alternative nitrogenase usage we also measured environmental Mo, Fe, and V concentrations at our sampling sites. Our results suggest that alternative nitrogenases are widely distributed in sequenced genomes and that diverse and active assemblages of organisms encoding these genes are found in coastal environments.

## Materials and Methods

### Genome Searches

**FIGURE 1:**
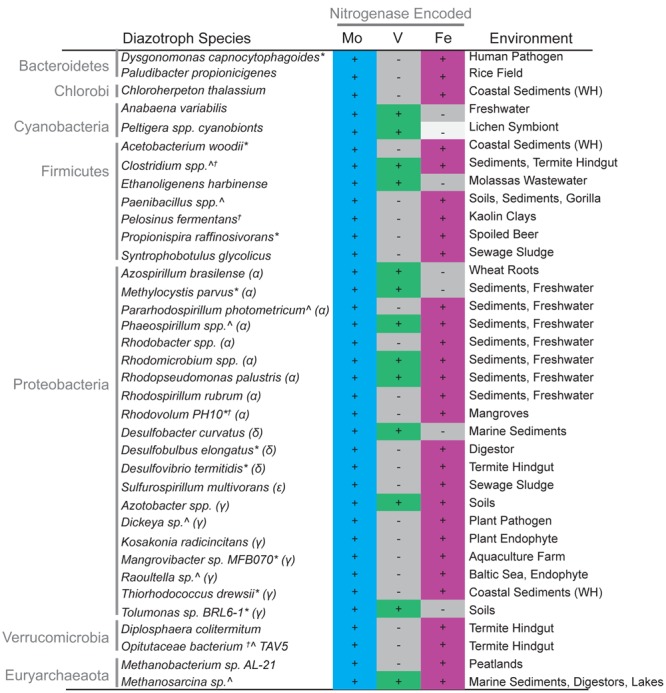
**Presence of nitrogenase isozyme genes in organisms with sequenced genomes and locations of isolation.** + indicates the presence of genes encoding Mo-nitrogenase, Fe-only nitrogenase, or V-nitrogenase as dictated by the minimum gene set (see Materials and Methods). - indicates organisms lacking genes for Mo, Fe-only, or V-nitrogenase. *Peltigera* spp. cyanobionts are shown in white as genomes are not available and therefore absence is not definitive. For brevity, the maximum gene set found in a give genus is shown, other members may encode different variations (Supplementary Table S1). α, Alphaproteobacteria; δ, Deltaproteobacteria; 𝜀, Epsilonproteobacteria; γ, Gammaproteobacteria. ^∗^ Genera discovered in this study not previously known to encode alternative nitrogenases; Λ genera known previously to encode alternative nitrogenases for which new species were added by this study. ^†^ Organisms lacking either NifN, NifE, or both. WH: Woods Hole, MA.

**FIGURE 2:**
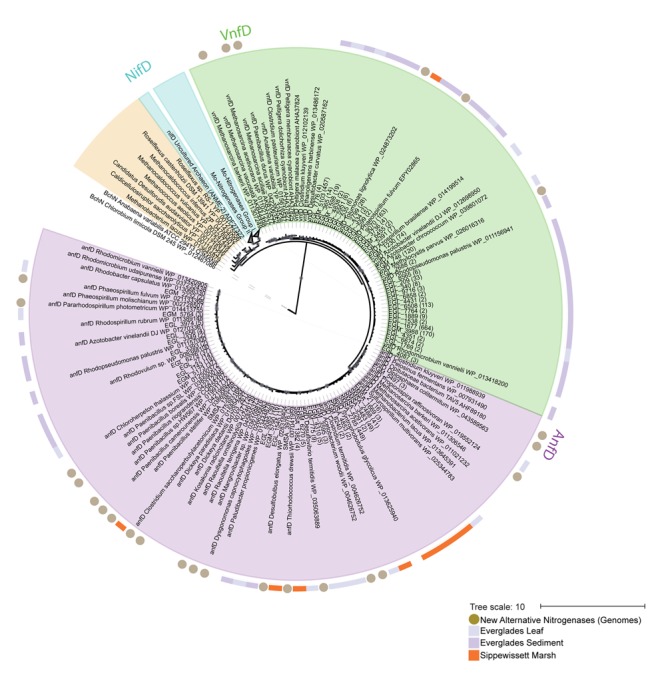
**Maximum likelihood phylogeny of Anf/Vnf/NifD protein sequences, focusing on alternative nitrogenase genes and amplicons.** A reference tree was built using complete NifD, AnfD, VnfD, and uncharacterized sequences. Operational taxonomic units (OTUs) recovered by this study (97% clustering, with >1 sequences per OTU) were placed using pplacer (Matsen et al., 2010). The number of sequences in each OTU is shown in parentheses. ANME-2 clusters with uncharacterized nitrogenases but has been recently proposed to be a Mo-based nitrogenase (McGlynn et al., 2013). Organisms with sequenced genomes for which alternative nitrogenases have been newly proposed in this study are indicated with circles in outer ring. Sampling locations for OTUs are indicated by outer ring colors: purple, Everglades leaves; light purple, Everglades sediments; orange, Sippewissett Marsh. Gray circles indicate aLRT estimates (SH > 0.6) for reference tree. An un-collapsed tree is shown in Supplementary Figure S1. EGL_2799 represents an OTU found in all environments, it was also the only V-nitrogenase OTU with >1 representative from Sippewissett Marsh and is shown in orange in the outer circle for emphasis.

We conducted BLASTP searches in NCBI (October 2015) using AnfG (Avin_48980) and VnfG (Uniprot: C1DI24) sequences from *Azotobacter vinelandii* as queries. Genomes for organisms with hits (<e^-20^) were downloaded from GenBank; organisms in which alternative nitrogenases were previously identified by (Dos Santos et al., 2012) were excluded. *A. vinelandii* NifHDKENB (Uniprot: P00459, P07328, P07329, P08293, P10336, P11067), AnfHDKG (Avin_49000, Avin_48990, Avin_48970, Avin_48980) and VnfHDKG (Uniprot: P15335, P16855, P16856, C1DI24) were used to check for the presence of alternative nitrogenases. To confirm the identity of sequences as alternative nitrogenases as opposed to protochlorophyllide or chlorophyllide reductases (which are related see Boyd and Peters, 2013) we also conducted BLASTP searches using BchLNXY sequences from *Rhodobacter capsulatus* (Uniprot: D5ANS3, P26164, P26177, P26178). Organisms were considered to have putative alternative nitrogenases if they had gene suites with best hits (based on *e*-value) to NifDKENB in addition to Anf/VnfDKG and at least one H protein. A few organisms lacked NifE, NifN, or both and are indicated with ^†^ in **Figure 1**. Reported habitats for each organism were determined based on the original isolation paper if possible or from Bergey’s Manual (Garrity, 2006) and the relevant culture collection (Supplementary Table S2). Designations as Fe-only or V-nitrogenase genes were made based on best BLAST and verified by placement on a phylogenetic tree (**Figure 2** and Supplementary Figure S1).

### Environmental Sample Collection

Samples were collected in June 2014 from a total of three sites (1) Everglades sediments (2) Everglades leaf litter (3) and Sippewissett Marsh sediments. Sample collection in the Everglades took place in a mangrove stand near West Lake Pond in Everglades National Park, FL, USA. Samples were collected from *Spartina alterniflora* dominated sediments in Great Sippewissett Marsh (Falmouth, MA, USA). Sediments in both Sippewissett Marsh and the Florida Everglades were loose and did not allow for the use of a soil corer. Instead, ∼50 ml of the top 5–10 cm of soil was collected with a UV-sterilized spatula. Leaf litter was collected directly into sterile bags. Samples for DNA extraction were collected in duplicate and stored in UV-sterilized conical tubes (sediments) and bags (leaves) and frozen at -20°C. For metal analyses, duplicate samples were collected in acid cleaned polypropylene tubes. Samples for the acetylene reduction assay were collected directly into acid cleaned glass incubation jars. All samples from a given site were obtained on the same day. To extract porewater, sediments were centrifuged and the supernatant was decanted, filtered (0.22 μM), and frozen (-20°C) for later analysis.

### Nucleic Acid Extraction and PCR Amplification

DNA extraction and polymerase chain reaction (PCR) amplification generally followed the protocols suggested by Zehr and Turner (2001). DNA was extracted using the PowerSoil DNA Isolation kit (MO BIO Laboratories Inc., Carlsbad, CA, USA) and purified with the MO BIO PowerClean DNA Clean-Up Kit. Reagents were 0.22 μm filtered (except for PowerClean Solution #2) and extractions were performed in a UV-Hood fitted with a HEPA filter. Pipettors, racks, and hood surfaces were cleaned with Decon^TM^ ELIMINase (Decon Labs, King of Prussia, PA, USA). Racks and plasticware were UV-Sterilized before usage. Additionally, blank extractions were conducted alongside sample extractions and were tested for amplification of target genes (see below).

For PCR amplification, DNA extracts from duplicate samples were pooled. *Vnf/anfD*, and *nifD* sequences were amplified using a nested PCR protocol with primers from (Bellenger et al., 2014). For *nifD:* the first round of PCR amplification was conducted using nifD820F: 5′-CAC TGC TAY CGB TCG ATG AAC TAC-3′ and nifD1389R: 5′-GAT GTC RCG SGC GAA GAT-3′ followed by a second round of amplification using nifD820F and nifD1331R: 5′-CAG GAG TGC ATY TGV CGG-3′, yielding a final amplicon of 512 base pairs. For *anf/vnfD* an initial PCR amplification was conducted using vnfD_anfD548F 5′-TSA AYA TCG SCT GGR TSA-3′ and vnfD_anfD1337R 5′-GCG TTR TAV ATR TCK CGS GC-3′ followed by a second round of amplification using vnfD_anfD548F and vnfD_anfD1291R 5′-TGT ANG GRC CRT TGT GRT A-3′, with a final amplicon length of 744 bp. Numbers used in primers correspond to sequence positions in *A. vinelandii nifD* and *anf/vnfD.*

PCR reactions (25 μl) were conducted using final concentrations of 2U Expand High Fidelity DNA Polymerase (Roche Applied Science, Indianapolis IN, USA) per reaction, 200 μM dNTPs, and 500 nM forward and reverse primer. 2 μl of DNA template (24–30 ng DNA) or control extract was added per reaction. PCR conditions for all reactions were: 94°C for 2 min, followed by 31 cycles of 94°C for 15 s, 55°C for 30 s, and 72°C for 2 min, with a final elongation cycle of 72°C for 7 min. For clone libraries, amplicons were verified using an ethidium bromide stained agarose gel and imaged using a UV transilluminator. To minimize DNA damage, PCR products used for PacBio sequencing were verified using agarose stained with SYBR gold (Invitrogen, Carlsbad, CA, USA) and visualized with a blue light transilluminator. PCR products were gel purified using the QIAquick gel extraction kit, (Qiagen, Valencia, CA, USA). Control extractions did not show visible bands, nonetheless, the region of the gel where the band was expected to occur was also excised and purified to confirm the absence of contaminating nitrogenase sequences.

### Clone Library Construction and PacBio Sequencing

A small initial clone library was created for each site and amplicon (Sippewissett Marsh clones were added to those from Zhang et al., 2016). Clone library sizes were as follows: Everglades Sediments: 77 *anf/vnfD*, 91 *nifD;* Everglades leaves: 92 *anf/vnfD*, 80 *nifD; Sippewissett Marsh sediments*: 79 *anf/vnfD*, 72 *nifD.* Unique clone library sequences have been deposited in GenBank (KY441123-KY441400). Purified products were cloned using the TOPO vector (Invitrogen, Life Technologies, Grand Island, NY, USA) and One-Shot TOP10 (Invitrogen, Life Technologies) chemically competent *Escherichia coli.* Plasmid DNA from overnight cultures was purified with the QIAprep Miniprep kit (Qiagen, Valencia, CA, USA) and unidirectional Sanger DNA sequencing was conducted at Macrogen (Macrogen USA, New York, NY, USA). Additionally, 37 *nifD* and 36 *anf/vnfD* clones from control DNA extractions were examined (reported previously, Zhang et al., 2016). Only 1 *nifD* and 1 *anf/vnfD* sequence were found. Sequences with 100% identity to those found in controls were excluded from all further analysis.

We used single-molecule real-time (PacBio) sequencing to gain further insight into the distribution of alternative nitrogenase amplicons in the environment. In PacBio sequencing, linear DNA fragments are ligated to hairpin adapters. DNA is then incubated with a polymerase as well as fluorescently labeled dNTPs in a specialized zero-mode waveguide and the DNA sequence is determined using sequencing-by-synthesis (Eid et al., 2009). When applied to short amplicons such as those in our study, the DNA polymerase is able to synthesize the entire amplicon several times. For PacBio sequencing, *nifD* and *anf/vnfD* amplicons were pooled by site with a target of 33% *nifD* and 66% *anf/vnfD* based on copy number (allowing for equal copies of *nifD, anfD*, and *vnfD*). Approximately 1000 ng PCR product per site was used. In addition to PCR amplicons, we also included a synthetic (gBlocks gene fragment, Integrated DNA technologies, Coralville, IA, USA) internal standard consisting of a 600 bp randomly generated sequence (60% GC), which was unrelated to nitrogenases and was added to samples at a target of 1% (based on total amplicon copies, Supplementary Figure S3). Library prep and sequencing were performed at the University of Maryland Institute for Genome Sciences using PacBio RS II, with 1 × 240 min movie per sampling site (pooled amplicons) and P6-C4 chemistry.

### Sequence Analysis, Operational Taxonomic Unit (OTU) Clustering and Phylogeny

PacBio data processing was adapted from (Schloss et al., 2016) with modification for protein coding genes. Consensus sequences were assembled from raw PacBio sub-reads using a cutoff of 90% accuracy and a minimum of two passes as well as a size selection for amplicons > 450 bp. These consensus sequences were blasted (BLASTX) to a curated alignment of Anf/Vnf/NifD protein sequences from clone libraries and sequenced genomes. Sequences without hits to this database (*e*-value cutoff of *e*^-5^) were discarded. Sequences were then screened for homopolymers (>10 discarded) and ambiguous bases (>0 discarded) using Mothur 1.35.1 (Schloss et al., 2009). Chimeric sequences were detected and removed using UCHIME (uchime_ref, Edgar et al., 2011), with an *anf/vnf/nifD* alignment as a reference. Nucleotide sequences were then translated to proteins and sequences with stop codons were discarded. Sequences were aligned using MUSCLE (Edgar, 2004) and trimmed to a uniform length of 458 bp, with any remaining shorter sequences discarded. As a final chimera removal step, *de novo* clustering was performed using USEARCH (cluster_otus, Edgar, 2010) and any identified chimeras were removed. Operational taxonomic unit (OTU) clustering and calculation of Shannon and Chao1, indices as well as re-sampling were performed using Mothur. Further data analysis and plotting was performed in R (R Development Core Team, 2012). OTU clustering of recovered internal control sequences was performed as described above, with a few modifications: referenced based chimera removal was not performed (due to the lack of an external reference alignment) and the requirement for full translation was not enforced (as the sequences did not correspond to a protein coding gene).

For phylogenetic trees, a reference protein alignment (MUSCLE) and tree (PhyML, Guindon and Gascuel, 2003) were built using full length Anf/Vnf/NifD proteins from organisms with sequenced genomes using the WAG model (Whelan and Goldman, 2001). Representative sequences from alternative nitrogenase OTUs (97% clustering) with >1 sequence per OTU were then translated and added to the tree using pplacer (Matsen et al., 2010). Missense insertions found during translation (EGL_1538, EGL_4351, EGL_1769, EGL_6858, EGL_4431, SMSA_1669 and SMSA_4853) were excluded in translations used for phylogenies. Tree graphics were produced using iTOL (Letunic and Bork, 2011).

Conserved cysteine (αCys275) searches were conducted using PacBio amplicon sequences that were screened for homopolymers, ambiguous bases, and contaminants. Full translation of the sequence without stop codons was not required. We searched for the presence of conserved ‘CAR’ residues in 10,325 sequences identified (best BLAST) as alternative nitrogenases. Sequences were then trimmed to the ‘--V--CAR---Y’ motif and any sequences lacking the entire motif (short sequences) were removed, as were any singletons. This yielded a total of 8,169 sequences. Anf/vnfD proteins from clone libraries and sequenced genomes were also examined as described above. The frequency of each residue was visualized using Weblogo: http://research4.dfci.harvard.edu/cvccgi/blocklogo/blocklogo.pl (Schneider and Stephens, 1990; Crooks et al., 2004).

### Acetylene Reduction and Isotopic Acetylene Reduction Assays

Acetylene reduction assays (ARA) were performed in 450 ml gas tight jars fitted with rubber septa under ∼10% v/v acetylene headspace. Five experimental replicates and five (no-acetylene) controls were incubated outside in 2–3 inches of ambient temperature water and kept in the shade (∼30% PAR). Acetylene reduction rates were checked by withdrawing samples of gas headspace at 0, 15, 18, 21, and 24 h (sediments) or 0, 15, 21, 24, 39, and 45 h (leaves). Ethylene concentrations were detected using a gas chromatograph with a flame-ionization detector (Shimadzu GC-8A, Shimadzu Corporation, Kyoto, Japan). Acetylene reduction rates were calculated from a linear regression of the first three time points. Control samples showed negligible (<2 ppmv) acetylene production.

Full ISARA methods are described by Zhang et al. (2016). Briefly, this technique uses known differences in the *in vivo* ^13^C fractionation of acetylene reduction to ethylene associated with Mo-, Fe-only, and V-nitrogenases to determine contributions from these isozymes in environmental samples. Headspace samples for ISARA were collected at the end of experiments and stored in evacuated, 200 ml serum bottles for later analysis. Isotopic analysis of the δ^13^C of ethylene and acetylene was conducted using gas chromatography-combustion-isotope ratio mass spectrometry (Thermo Scientific GC Isolink - Delta V Advantage Isotope Ratio Mass Spectrometer with a Conflo IV). Raw isotope values were calibrated to V-PDB scale using methane isotope standards (range -38.3 to -23.9‰, methane 1 from Indiana University; Tiso and Hiso from Isometric Instruments). The fractional contributions of alternative nitrogenases to acetylene reduction were calculated as in Zhang et al. (2016).

### Trace Metal Measurements

For trace metal analysis, ∼0.5–1 g dry soil or leaf sample was digested in a Teflon tube with trace metal grade HNO_3_ (65 w/w%, Optima, Douglas, GA, USA) using a MARS 6 digester (CEM, Matthew, NC, USA). Water samples were not digested. Samples were run on an Inductively Coupled Plasma Mass Spectrometer (Element 2; Thermo Finnigan, Somerset, NJ, USA) at medium resolution. Element counts were normalized to internal (Indium) standards and Mo, Fe, and V concentrations were determined using standard curves for each element.

## Results

### Alternative Nitrogenase Genes in Sequenced Genomes

In order to understand the taxonomic distribution of alternative nitrogenases, we searched for NifHDKENB, as well as AnfHDKG and VnfHDKG protein sequences in publically available genomes (GenBank, June 2015). This work builds on a previous study (Dos Santos et al., 2012) in which genome searches identified a number of potential diazotrophs and yielded a minimum gene set consisting of *nifHDKENB* required for nitrogen fixation. In our study, putative alternative nitrogenases were discovered in 25 species not previously known to encode these genes (**Figure 1**, Supplementary Figure S2 and Tables S1, S2). These were in genera in which alternative nitrogenases have been well documented such as *Methanosarcina* and *Clostridium*. However, we also found alternative nitrogenases in 10 genera where these genes have yet to be reported. This finding adds to the 29 species and 25 genera with previously published documentation of alternative nitrogenases (Supplementary Table S1).

We used phylogenetic reconstructions to further verify the metal-center used in these recovered nitrogenase sequences. The evolutionary history of nitrogenase isozymes has been a topic of extensive study (Raymond et al., 2004; Boyd et al., 2011a,b; Boyd and Peters, 2013; McGlynn et al., 2013). While most authors conclude that single gene phylogenies provide inconsistent results as to the evolution history of the isozymes, Anf/Vnf/NifD phylogenies do successfully predict isozyme metal-centers with a few noted exceptions (McGlynn et al., 2013). Phylogenetic placement (**Figure 2** and Supplementary Figure S1) of NifD and Anf/VnfD protein sequences recovered from each of the organisms in this study suggests that they all encode both canonical (NifD) and alternative (AnfD or VnfD) nitrogenase proteins, which cluster with those from other established diazotrophs.

In order to understand the types of environments that might harbor alternative nitrogenases, we searched for the location from which each organism was isolated. Original isolation papers were used whenever possible (Supplementary Table S1). Organisms encoding alternative nitrogenases (both previously reported and newly discovered) spanned a range of environments (**Figure 1** and Supplementary Table S2). The majority of organisms with alternative nitrogenases were isolated from soils/sediments, freshwater or anthropogenic environments such as wastewater treatment and agricultural sites. However, a handful seemed to be associated with macroscopic organisms either as pathogens or symbionts.

### Recovery of Alternative and Canonical Nitrogenase Amplicons from Environmental Samples

Sequenced genomes searches suggested that organisms with alternative nitrogenases are common in sediments (**Figure 1**). Based on this finding, we explored alternative nitrogenase diversity in three sampling sites (1) sediments from the Florida Everglades, (2) leaf litter from the Florida Everglades (3) sediments from Sippewissett Marsh (Falmouth, MA, USA). In the Everglades, sediments and leaves were collected from a mangrove stand. In Sippewissett Marsh, sediment samples were collected from *S. alterniflora* dominated sediments (details found in Zhang et al., 2016). The presence of nitrogenase isozyme sequences was verified using clone libraries of *nifD* (72–91 clones per sampling site) as well as *anf/vnfD* (77–92 clones per sampling site) amplicons.

Nitrogenase diversity was more deeply explored using high throughput sequencing of *nifD* and *anf/vnfD* PCR amplicons. Here, we took advantage of the long sequence reads of single-molecule real time sequencing (PacBio, Eid et al., 2009) which allowed for the generation of a library of nearly full-length amplicon sequences. Consensus sequence filtering (90% accuracy with a minimum of two passes), size selection (>450 bp) and BLASTX screening for nitrogenases yielded 23,889 *anf/vnf/nifD* sequences across Everglades leaf, Everglades sediments, and Sippewissett Marsh sediments, all of which have been deposited in the sequence read archive at GenBank (BioProject ID: PRJNA360640). Further diversity analysis was conducted using a more stringent screening constraint that required all sequences to translate without stop codons. This produced a smaller, higher quality data set consisting of 841 *anfD*, 2,026 *vnfD*, and 3,972 *nifD* sequences (356, 659, and 2,683 unique OTUs, respectively). Prior to this study, a total of 79 *anfD* and 80 *vnfD* and DNA sequences were available in GenBank (based on text searches for ‘anfD’ and ‘vnfD’ in the GenBank nucleotide database, June 2015). These additional sequences greatly increase the number of potential alternative nitrogenase sequences available.

### Validation of High Throughput Sequencing Results

**FIGURE 3:**
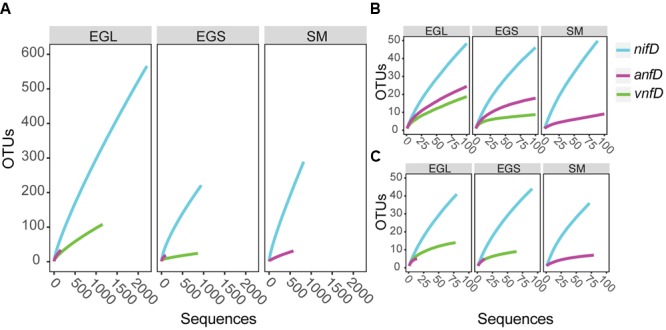
**Rarefaction curves for *nifD, anfD*, and *vnfD* amplicons clustered (97%) at different sampling sites. (A)** Full data set from PacBio sequencing **(B)** truncated PacBio dataset **(C)** clone library data. EGL, Everglades leaf; EGS, Everglades sediments; SM, Sippewissett Marsh.

PacBio sequencing has been widely used to sequence genomes and more recently has been applied to 16S rRNA genes (Fichot and Norman, 2013; Mosher et al., 2014; Schloss et al., 2016). However, to our knowledge, this is the first study to apply PacBio sequencing to functional genes. Accordingly, we took a number of steps to validate our sequencing results, including a very stringent screening process for the data (see above). In addition, we included a 600 bp internal standard in our samples to assess sequencing error (see Materials and Methods). The number of spurious OTUs expected based on clustering of this standard was calculated and compared to our sample rarefaction curves (Supplementary Figures S3A,B). Sample rarefaction estimates were consistently higher than those expected due to sequencing error alone (see supplement for further discussion). In addition, rarefaction curves were constructed using sequences from our clone library dataset (generated with Sanger sequencing) and found to be largely consistent with those predicted by our PacBio dataset (**Figures 3B,C**).

### Environmental *nifD, anfD*, and *vnfD* Diversity

Operational taxonomic unit clustering of DNA sequences at 97% (average neighbor) and rarefaction analyses indicated that numerous *nifD, anfD*, and *vnfD* OTUs were present in all environments and that *nifD* diversity was typically higher than that of *anfD* and *vnfD* (**Figure 3A**). This result was also seen at 95% clustering levels (Supplementary Figure S4). Differences in the isozyme pools from each site were further explored using diversity indices (**Table 1A**). As these indices can be sample-size dependent, we also conducted calculations using an evenly re-sampled dataset (**Table 1B**). Shannon and Chao1 diversity estimates as well as observed OTU counts (*S*_obs_) were all higher for *nifD* than for *anfD* or *vnfD* amplicons regardless of which dataset was used (**Tables 1A,B**).

**Table 1:** Diversity indices calculated for *nifD, anfD*, and *vnfD* in all sampling sites using **(A)** full dataset **(B)** a dataset resampled to 100 sequences per amplicon per site.

Site	Gene	Sampling Depth	*S*_obs_ (97%)	Chao1 (*SE*)	Shannon	% (*S*_obs_)	% (Chao1)
**(A) Diversity indices**

EGL	*nifD*	2207	**566**	**2822 (390)**	4.36	80	85
	*anfD*	161	34	126 (57)	2.51	5	4
	*vnfD*	1153	108	378 (97)	2.09	15	11
							
EGS	*nifD*	944	221	781 (146)	4.04	84	91
	*anfD*	102	18	21 (3)	2.18	7	2
	*vnfD*	862	24	54 (23)	1.54	9	6
							
SM	*nifD*	821	289	2249 (552)	**4.47**	**90**	**95**
	*anfD*	578	31	108 (50)	0.99	10	5
	*vnfD*	11	2	2 (0)	0.30	<1	<1
**(B) Diversity indices, Resampled dataset**

EGL	*nifD*	100	**52**	**353 (170)**	3.41	58	79
	*anfD*	100	20	38 (15)	2.27	22	8
	*vnfD*	100	18	57 (30)	1.76	20	13
							
EGS	*nifD*	100	47	205 (87)	3.33	63	85
	*anfD*	100	18	21 (3)	2.20	24	9
	*vnfD*	100	10	16 (7)	1.67	13	7
							
SM	*nifD*	100	51	146 (47)	**3.51**	**85**	**86**
	*anfD*	100	9	24 (14)	0.87	15	14
	*vnfD*	100	–	–	–	–	–

*The amplicon and site combination predicted to be most diverse by a given index are shown in bold. % *S*_obs_: percent of total OTUs observed corresponding to a given amplicon per site. % Chao1: percent of Chao1 predicted OTUs belonging to a given amplicon per site. SE: standard error, S*_obs_*: number of predicted OTUs at 97% clustering. –: not included due low sample size (11 sequences). EGL, Everglades leaf; EGS, Everglades sediment; SM, Sippewissett Marsh. Percentages do not always sum to 100 due to rounding.*

**FIGURE 4:**
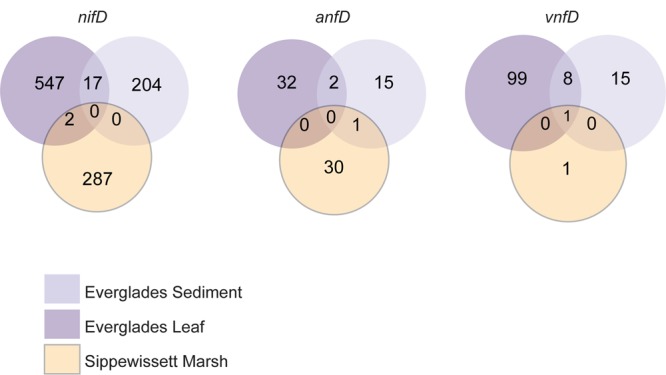
**Nitrogenase amplicon OTU overlap between sampling sites for sequences clustered at 97%.** EGL (purple), Everglades leaf; EGS (light purple), Everglades sediments; SM (orange), Sippewissett Marsh.

Recovered nitrogenase OTUs from all three amplicons were also site specific, with very little overlap between sites and only 1 OTU that was found in all three sites (**Figure 4**). While estimates of *nifD* diversity were fairly comparable across all three sampling sites (Shannon diversity of 3.33–3.51 in the evenly re-sampled dataset), *anfD* and *vnfD* diversity was typically higher in Everglades (particularly Everglades leaf litter) samples (Shannon diversity of 2.20–2.27 as opposed to 0.87 for *anfD*). In addition, *vnfD* amplicons were almost entirely absent from the Sippewissett Marsh dataset, with only 11 sequences recovered (compared to 1,153 and 862 from Everglades leaf and sediments samples, respectively). One reason for the lack of *vnfD* amplicon recovery in Sippewissett Marsh could be that our current primer set does not capture the full range of taxonomic variation in this environment.

**FIGURE 5:**
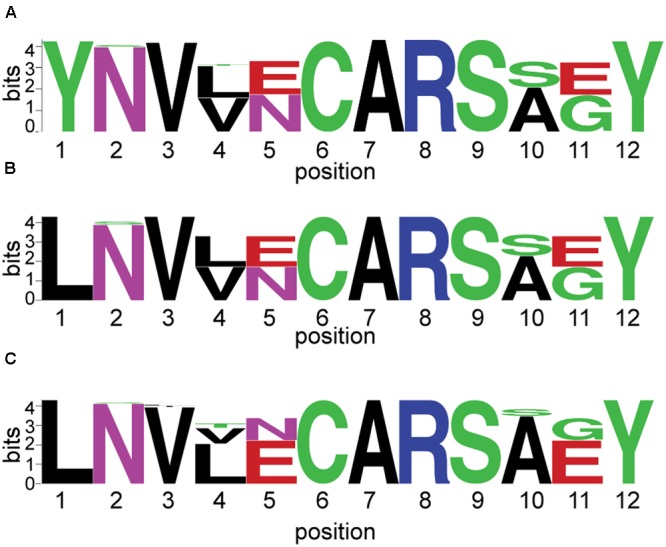
**Sequences flanking conserved αCys275 in alternative nitrogenase amplicons and full length sequences.** αCys275 is shown at position 6 on the *x*-axis. The relative frequency of each residue is indicated by its height on the *y*-axis. This analysis was conduced for: **(A)** 8,169 amplicons sequenced using PacBio technology; **(B)** 119 amplicons sequenced using Sanger Sequencing (clone libraries); and **(C)** 62 sequences from isolates with sequenced genomes.

Finally, representative sequences from *anfD* and *vnfD* OTUs were translated and placed onto a phylogenetic tree (**Figure 2** and Supplementary Figure S1), confirming their homology to alternative (as opposed to canonical or uncharacterized) nitrogenase genes. The functionality of these amplicons was further investigated through the analysis of amino acid residues surrounding the metal cofactors. Previous studies have reported that a conserved cysteine residue (αCys275) along with a histidine (αHis442, numbering based on *A. vinelandii* sequences) are needed to serve as ligands for the FeMo cofactor in *nifD* sequences, and that these residues are conserved in alternative nitrogenases (reviewed in Eady, 1996; Howard and Rees, 1996). For this analysis we conducted a broad search of our PacBio dataset, recovering 8,169 *anf/vnfD* amplicons with αCys275 (**Figure 5**; αHis442 falls within the primer sequence and was not examined). All sequences found in clone libraries as well as sequenced genomes also encoded αCys275. The amino acids seen in the region flanking αCys275 were consistent with those reported in previous studies using sequenced genomes (Glazer and Kechris, 2009; Dos Santos et al., 2012; Howard et al., 2013). Overall, our study greatly increases the number of *anfD* and *vnfD* sequences available for further investigations of the environmental distribution (**Figures 3**, **4**), phylogeny (**Figure 2** and Supplementary Figure S1) and function (**Figure 5**) of nitrogenase isozymes.

### Nitrogen Fixation and Activity of Alternative Nitrogenases in Everglades Samples

**FIGURE 6:**
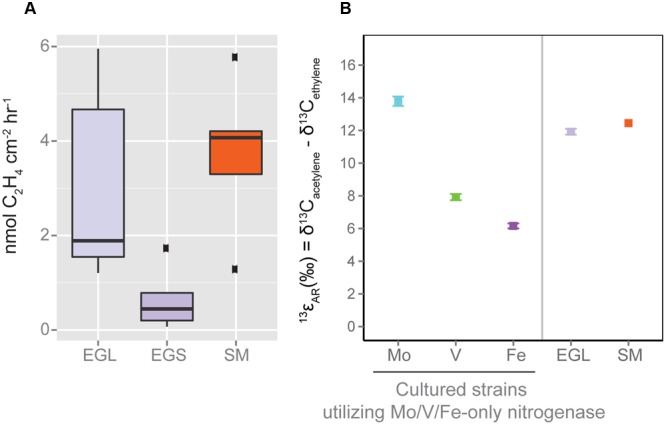
**Nitrogen fixation and contribution of alternative nitrogenases as determined by (A)** Acetylene Reduction Assay and **(B)** Isotopic Acetylene Reduction Assay (ISARA). SM data and reference Mo, Fe, and V-nitrogenase ^13^𝜀_AR_ values are from (Zhang et al., 2016). ISARA values are calculated using the three most active EGL and SM samples from **(A)**. EGL sample ^13^𝜀_AR_ values are suggestive of a mixture of alternative and canonical nitrogenase use. ISARA error bars are standards errors of three replicate incubations. EGL, Everglades Leaf; EGS, Everglades Sediment; SM, Sippewissett Marsh.

Acetylene reduction assays conducted on both leaves and sediments from the Everglades showed active nitrogen fixation (**Figure 6A**). Alternative nitrogenase isozymes contributions were tested using the newly developed ISARA technique (Zhang et al., 2016), which distinguishes between canonical and alternative isozymes based on established kinetic isotope effects of acetylene reduction to ethylene (^13^𝜀_AR_ = δ^13^C_acetylene_ - δ^13^C_ethylene_) for each nitrogenase isozyme: ^13^𝜀_Mo_ = 13.8 ± 0.3, ^13^𝜀_V_ = 7.9 ± 0.2, and ^13^𝜀_Fe_ = 6.2 ± 0.2 (errors are SE). ISARA measurements from the three most active leaf samples showed a ^13^𝜀_AR_ value (11.9 ± 0.2) significantly lower (*p* < 0.01) that of pure Mo-nitrogen fixation in cultures (**Figure 6B**). This value was also lower than reported for Sippewissett Marsh samples (^13^𝜀_AR_ = 12.5 ± 0.6, Zhang et al., 2016), although the difference was not significant, (*p* = 0.20). Sediments from the Everglades could not be measured because acetylene reduction rates were too low to produce the ethylene quantities needed for ISARA.

### Environmental Trace Metal Concentrations

**FIGURE 7:**
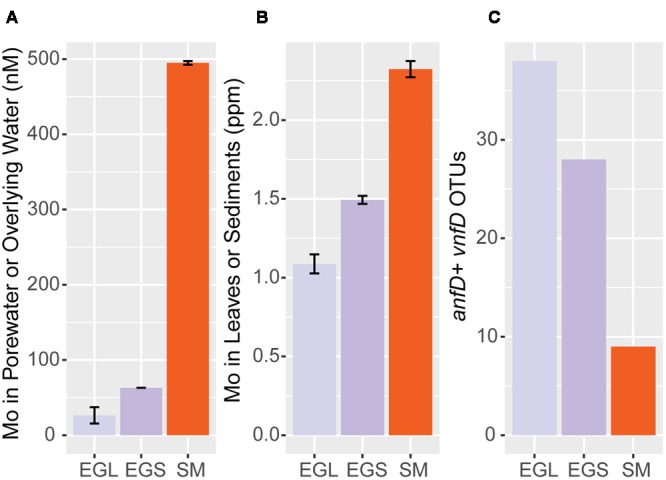
**Mo concentrations and *anf/vnfD* OTUs across sampling sites. (A)** Mo content in overlying water (EGL) and porewater (EGS, SM) **(B)** Mo content in leaves (EGL) and sediments (EGS, SM) **(C)** alternative nitrogenase OTUs (97%) recovered from 100 randomly sampled *anf/vnfD* amplicon sequences (**Table 1B**). EGL; Everglades leaf, EGS; Everglades sediment; SM: Sippewissett Marsh. Porewater Mo values are from (Zhang et al., 2016).

Previous research suggests that Mo-nitrogenases are more efficient than Fe-only and V-nitrogenases (Eady and Robson, 1984) and that Mo can repress expression of these enzymes (Jacobson et al., 1986; Walmsley and Kennedy, 1991). We examined whether Mo concentrations might vary between our different sampling sites. Mo, Fe, and V concentrations were measured in sediments, leaves, porewater, and overlying water from our sampling sites. Although all sampling sites were coastal, V and Fe concentrations were greater than those of Mo in leaf litter as well as sediments, as is consistent with a strong terrestrial influence (Supplementary Table S3, Wedepohl, 1995). However, Mo concentrations varied between sampling sites, in the order: Everglades leaf litter < Everglades sediment < Sippewissett Marsh sediment and Everglades overlying water < Everglades porewater < Sippewissett Marsh porewater (**Figures 7A,B** and Supplementary Table S3). When compared to our findings of *anfD* and *vnfD* OTUs, samples with lower Mo concentrations had higher numbers of *anfD* and *vnfD* OTUs (**Figure 7C** and **Tables 1A,B**).

## Discussion

### Taxonomic Distribution of Alternative Nitrogenase Genes

The distribution of nitrogen fixation genes in bacterial and archaeal genomes is patchy and complicated by duplication events and horizontal gene transfers (Raymond et al., 2004; Young, 2005; Boyd and Peters, 2013). Our survey of alternative nitrogenase genes reveals a similar pattern and is largely consistent with the current understanding of the distribution of these genes in isolates. As observed previously (Dos Santos et al., 2012), all organisms with alternative nitrogenases also encoded canonical Mo-nitrogenases. Accordingly, the classes found to harbor alternative nitrogenase sequences were a subset of those known to contain canonical nitrogen fixers. Alternative nitrogenase genes were found (both by this study and others) in α-, γ-, and δ/𝜀-proteobacteria. Interestingly, no alternative nitrogenase sequences have been recovered from the β-proteobacteria despite the presence of canonical nitrogen fixers in this class and the availability of numerous genomes in GenBank (Supplementary Figure S2). We also found that the number of organisms with putative alternative nitrogenases in a given taxonomic group increased with the number of available genomes (Supplementary Figure S2). As such, genome sequencing efforts should continue to reveal organisms with alternative nitrogenases and provide a broader understanding of the taxonomic distribution of these enzymes.

### Diversity and Overall Contribution of Alternative Nitrogenases

The overall role of alternative nitrogenases in nature remains an important and heretofore unanswered question. Our dataset of *nifD* and *vnf/anfD* amplicons from the same environment allows for the first comparison of alternative and canonical nitrogenase diversity. These calculations will need to be refined as better primers and more quantitative methods for the detection of alternative nitrogenases (such as qPCR) become available. Nonetheless, using Chao1 richness estimates from an evenly re-sampled dataset (**Table 1B**), we calculate that *vnfD* and *anfD* OTUs account for 21% of all nitrogenase OTUs in Everglades leaf samples, compared with 16% in Everglades sediment samples and 14% in Sippewissett Marsh samples (**Table 1B**).

Using an independent method, the ISARA, we report that alternative nitrogenases are active in the Everglades leaf samples. The ISARA method cannot distinguish between Fe-only and V-nitrogenase usage. However, by making the most conservative assumption of 100% Fe-only nitrogenase usage (which has the smallest ^13^𝜀_AR_, see Zhang et al., 2016) we calculate the fractional alternative nitrogenase contribution to acetylene reduction for leaf samples to be 24%. The fractional contribution found in Sippewissett Marsh was 18% (Zhang et al., 2016). This number provides a lower bound on activity and is likely to increase when converted to nitrogen fixation as the *R*-ratio (R = acetylene reduction/N_2_ fixation) is typically higher for Mo-nitrogenases as opposed to alternative nitrogenases (Bellenger et al., 2014). Together, our sequencing data as well as ISARA estimates place alternative nitrogenase diversity at 14–21% of total nitrogenase diversity and suggest that they can make a considerable (≥24%) contribution to nitrogen fixation.

### Possible Trace Metal Drivers of Alternative Nitrogenase Usage in Terrestrial Systems

Trace metals have been commonly proposed as regulators of nitrogenase isozyme usage. However, due to the paucity of information on environmental alternative nitrogenases, it remains unclear how widespread the connection between Mo concentrations and alternative nitrogenase diversity and activity may be. We found alternative nitrogenases in the genomes of organisms living in many different types of environments, ranging from sediments to insect guts. Alternative nitrogenase usage is likely shaped by very different factors across environments. In the termite hindgut, one of the few locations where alternative nitrogenase gene expression has been demonstrated *in situ*, researchers found little relationship between Mo concentrations and alternative nitrogenase gene expression (Noda et al., 1999). Studies of alternative nitrogenases in lichens also suggest the link between environmental trace metal concentrations and isozyme usage is not always straightforward (Darnajoux et al., 2014, 2016; Zhang et al., 2016). In our coastal samples, Mo concentrations were relatively high. Nonetheless, we recovered diverse assemblages of *anfD* and *vnfD* amplicons from all environments examined and found that environments with higher Mo concentrations had lower numbers of alternative nitrogenase OTUs (**Figures 7A–C**). This finding raises the possibility that isozyme diversity and activity can be connected to environmental trace metal concentrations. The strength of this relationship and whether it is the exception, the norm, or just one of many scenarios for alternative nitrogenase usage remains to be seen.

## Conclusion

In recent years, our understanding of the N cycle has been expanded by the inclusion of both new environments for nitrogen fixation as well as new organisms performing this task. While Mo-nitrogenases have been well studied in the environment, knowledge of alternative nitrogenases has remained almost non-existent, with very few sequences available and little information about their distribution in nature. Our data provide the most extensive set of environmental alternative nitrogenase amplicon sequences to date and demonstrate that alternative nitrogenases are present and active in coastal environments. The organismal ‘choice’ to use one nitrogenase as opposed to another is likely complex and further studies will be needed to fully understand this phenomenon. However, our trace metal results lend support to the hypothesis that Mo availability influences isozyme usage. The increased *anfD* and *vnfD* sequence resolution provided in this study will help to facilitate future investigations, aiding in improvement of current primer sets as well as the development of more quantitative methods such as qPCR. While many questions remain regarding the drivers of alternative nitrogenase use, our findings emphasize the importance of a previously overlooked group of nitrogen fixing enzymes and suggest that trace metal controls on nitrogen fixation may be more complex than previously thought.

## Author Contributions

DM, XZ, AK, and FM designed the experiments. DM, XZ, and AK performed experiments, XZ and DM analyzed the data. DM, XZ, AK, and FM co-wrote the paper.

## Conflict of Interest Statement

The authors declare that the research was conducted in the absence of any commercial or financial relationships that could be construed as a potential conflict of interest.
