# Supplementary material for: Diversity and Activity of Alternative Nitrogenases in Sequenced Genomes and Coastal Environments
Source: Front Microbiol. 2017 Feb 28;8:267. doi: 10.3389/fmicb.2017.00267 (PMC5328986; doi:10.3389/fmicb.2017.00267)
Supplement: Supplementary file 1 [file Table_1.docx]

**Supplementary Table S1**. Accession numbers for genomes searched in this study and those that were previously reported.

| Species | NifB | NifE | NifN | NifD  AnfD/VnfD | NifH^^^  AnfH/VnfH | NifK  AnfK/AnfK | AnfG/VnfG |
| --- | --- | --- | --- | --- | --- | --- | --- |
| Bacteroidetes |  |  |  |  |  |  |  |
| *Dysgonomonas capnocytophagoides* DSM22835  [NZ_AUFL00000000.1](http://www.ncbi.nlm.nih.gov/nuccore/651642351) | WP_035331502.1 | WP_026626161.1 | WP_026626162.1 | WP_051290673.1  WP_026625683.1 | WP_026626156.1  WP_026625686.1 | WP_026626160.1  WP_026625681.1 | WP_026625682.1 |
|  |  |  |  |  |  |  |  |
| Firmicutes |  |  |  |  |  |  |  |
| *Acetobacterium woodii* DSM1030  [NC_016894.1](http://www.ncbi.nlm.nih.gov/nuccore/NC_016894.1) | WP_014355185.1^†^ | WP_014355186.1 | WP_014355185.1^†^ | WP_014355188.1  WP_014355095.1 | WP_014355191.1  WP_014355098.1 | WP_014355187.1  WP_014355093.1 | WP_014355094.1 |
| *Clostridium pasteurianum* BC1  [NZ_JPGY00000000.1](http://www.ncbi.nlm.nih.gov/nuccore/NZ_JPGY00000000.1) | WP_015616534.1^†^ | WP_041711555.1 | WP_015616534.1^†^ | WP_015616537.1  WP_015616547.1 | WP_015616537.1  WP_015616548.1  WP_015617187.1 | WP_015616536.1  WP_015616545.1 | WP_015616546.1 |
| *Clostridium saccharoperbutylacetonicum* N1-4 (HMT)  [NC_020291.1](http://www.ncbi.nlm.nih.gov/nuccore/NC_020291.1) | WP_015393161.1^†^ | WP_015393160.1 | WP_015393161.1^†^ | WP_015393158.1  WP_015393168.1 | WP_015393155.1  WP_015393167.1 | WP_015393159.1  WP_015393170.1 | WP_015393169.1 |
| *Clostridium termitidis* CT1112  [NZ_AORV00000000.1](http://www.ncbi.nlm.nih.gov/nuccore/475994024) | WP_004626538.1^†^ | WP_004626447.1 | WP_004626538.1^†^ | WP_004626464.1  WP_004626752.1 | WP_004626521.1  WP_004626804.1 | WP_004626425.1  WP_004626828.1 | WP_051066607.1 |
| *Paenibacillus borealis* DSM13188  [NZ_CP009285.1](http://www.ncbi.nlm.nih.gov/nuccore/NZ_CP009285.1) | WP_052429669.1 | WP_042217167.1 | WP_042217166.1 | WP_042217169.1  WP_042212386.1 | WP_042217170.1  WP_042212384.1 | WP_042217168.1  WP_042212390.1 | WP_042212388.1 |
| *Paenibacillus camerounensis* G4  NZ_CCDG000000000.1 | WP_042203060.1 | WP_042203056.1 | WP_042203055.1 | WP_042203059.1  WP_042198922.1 | WP_042180014.1  WP_042198924.1 | WP_042203057.1  WP_042198918.1 | WP_042198920.1 |
| *Paenibacillus durus* DSM1735  [NZ_CP009288.1](http://www.ncbi.nlm.nih.gov/nuccore/NZ_CP009288.1) | WP_042207945.1 | WP_042207941.1 | WP_042207940.1 | WP_042207943.1  WP_042206352.1 | WP_042207944.1  WP_042208220.1  WP_025692941.1 | WP_042207942.1  WP_042206353.1 | WP_025692943.1 |
| *Paenibacillus* sp. FSL H7-0357  [NZ_CP009279.1](http://www.ncbi.nlm.nih.gov/nuccore/NZ_CP009279.1) | WP_038595943.1 | WP_052092279.1 | WP_038595933.1 | WP_038595939.1  WP_038590013.1 | WP_038595941.1  WP_038590008.1 | WP_038595936.1  WP_038590019.1 | WP_038590016.1 |
| *Paenibacillus sp.* HW567  [NZ_ARFI00000000.1](http://www.ncbi.nlm.nih.gov/nuccore/486346089) | WP_019908851.1 | WP_019908855.1 | WP_019908857.1 | WP_019908853.1  WP_019911712.1 | WP_019908852.1  WP_019911713.1 | WP_019908854.1  WP_019911710.1 | WP_019911711.1 |
| *Propionispira raffinosivoran* DSM20765  [NZ_ARLE00000000.1](http://www.ncbi.nlm.nih.gov/nuccore/485071312) | WP_019553543.1^†^ | WP_019553542.1 | WP_019553543.1^†^ | WP_019553540.1  WP_019552124.1 | WP_019553537.1  WP_019552121.1 | WP_019553541.1  WP_026329190.1 | WP_019552125.1 |
| *Paenibacillus riograndensis* SBR5  [NZ_LN831776.1](http://www.ncbi.nlm.nih.gov/nuccore/NZ_LN831776.1) | CQR57928.1 | CQR57924.1 | CQR57923.1 | CQR57926.1  CQR55107.1 | CQR57927.1  CQR55106.1 | CQR57925.1  CQR55109.1 | CQR55108.1 |
| *Paenibacillus stellifer* DSM14472  [NZ_CP009286.1](http://www.ncbi.nlm.nih.gov/nuccore/NZ_CP009286.1) | WP_038698126.1 | WP_052098719.1 | WP_038698118.1 | WP_038698122.1  WP_038695269.1 | WP_038698124.1  WP_038695267.1  WP_038701478.1 | WP_038698120.1  WP_038695271.1 | WP_038700656.1 |
| *Pelosinus fermentans* R7  NZ_AKVN00000000.1 | WP_007936485.1 | WP_007933889.1 | --- | WP_007933893.1  WP_007931490.1 | WP_007933899.1  WP_007931496.1  WP_007936450.1 | WP_007933891.1  WP_007931488.1 | WP_007931489.1 |
|  |  |  |  |  |  |  |  |
| Proteobacteria (α) |  |  |  |  |  |  |  |
| *Azospirillum brasilense* sp 245  [NC_016617.1](http://www.ncbi.nlm.nih.gov/nuccore/NC_016617.1) | WP_052584333.1 | WP_014199505.1 | WP_014199506.1 | WP_014239785.1  WP_014199514.1 | WP_014239786.1  --- | WP_014239784.1  WP_014199516.1 | WP_014199515.1 |
| *Methylocystis parvus* OBBP  [NZ_AJTV00000000.1](http://www.ncbi.nlm.nih.gov/nuccore/NZ_AJTV00000000.1) | WP_016919567.1 | WP_016920356.1 | WP_016920355.1 | WP_016919584.1  WP_026016316.1 | WP_026016315.1  --- | WP_016919585.1  WP_016920363.1 | WP_016920362.1 |
| *Pararhodospirillum photometricum* DSM122  [NC_017059.1](http://www.ncbi.nlm.nih.gov/nuccore/NC_017059.1) | WP_051013987.1 | WP_051013892.1 | WP_051013891.1 | WP_014415840.1  WP_014413755.1 | WP_041797500.1  WP_041793880.1 | WP_014415839.1  WP_014413753.1 | WP_014413754.1 |
| *Phaeospirillum fulvum* MGU-K5 NZ_AQPH00000000.1 | WP_039853569.1 | WP_021131161.1 | WP_021131162.1 | WP_021130618.1  WP_021133366.1  WP_039851985.1 | WP_021132958.1  WP_021133367.1 | WP_021130619.1  WP_021133364.1  WP_021131155.1 | WP_021133365.1  WP_021131156.1 |
| *Phaeospirillum molischianum* DSM120  [NZ_CAHP00000000.1](http://www.ncbi.nlm.nih.gov/nuccore/381169400) | WP_002725216.1 | WP_002726540.1 | WP_002726538.1 | WP_002729386.1  WP_002728507.1 | WP_002728190.1  WP_002728509.1 | WP_002729388.1  WP_002728502.1 | WP_002728505.1 |
| *Rhodovulum* sp. PH10  [AKZI00000000.1](http://www.ncbi.nlm.nih.gov/nuccore/AKZI00000000.1) | WP_008391527.1 | --- | WP_008389210.1 | WP_008391589.1  WP_008388100.1 | WP_040622284.1  WP_008388099.1 | WP_008391590.1  WP_008388104.1 | WP_040620628.1 |
| *Rhodomicrobium udaipurense* JA643  [NZ_JFZJ00000000.1](http://www.ncbi.nlm.nih.gov/nuccore/739382109) | WP_037237957.1 | WP_037238743.1 | WP_037241489.1 | WP_037238711.1  WP_037232438.1 | WP_037238720.1  WP_037232440.1 | WP_037238708.1  WP_037232434.1 | WP_037232436.1 |
|  |  |  |  |  |  |  |  |
| Proteobacteria (δ) |  |  |  |  |  |  |  |
| *Desulfobacter curvatus* DSM 3379  [NZ_AREY00000000.1](http://www.ncbi.nlm.nih.gov/nuccore/NZ_AREY00000000.1) | WP_020587652.1 | WP_020586123.1 | WP_020587171.1* | WP_020586125.1  WP_020587162.1 | ---  WP_020587163.1 | WP_020586124.1  WP_020587160.1 | WP_020587161.1 |
| *Desulfobulbus elongates* DSM 2908  [NZ_JHZB00000000.1](http://www.ncbi.nlm.nih.gov/nuccore/654867648) | WP_028319049.1 | WP_028319047.1 | WP_028319048.1 | WP_028319057.1  WP_028318404.1 | WP_028319054.1  WP_035216184.1 | WP_028319058.1  WP_028318406.1 | WP_028318405.1 |
| *Desulfovibrio termitidis* Hl1  [NZ_AZAO00000000.1](http://www.ncbi.nlm.nih.gov/nuccore/737082685) | WP_035068241.1 | WP_035068240.1 | WP_051384659.1 | WP_035068236.1  WP_035063889.1 | WP_035068233.1  WP_035063892.1 | WP_035068237.1  WP_035063888.1 | WP_035069272.1 |
| *Sulfurospirillum multivorans* DSM 12446  [NZ_CP007201.1](http://www.ncbi.nlm.nih.gov/nuccore/NZ_CP007201.1) | WP_025344357.1 | WP_025344366.1 | WP_025344367.1 | WP_025344429.1  WP_025344783.1 | WP_025344427.1  --- | WP_025344430.1  WP_025344781.1 | WP_038533953.1 |
|  |  |  |  |  |  |  |  |
| Proteobacteria (γ) |  |  |  |  |  |  |  |
| *Dickeya paradisiaca* NCPPB 2511  [NZ_CM001857.1](http://www.ncbi.nlm.nih.gov/nuccore/NZ_CM001857.1) | WP_012764143.1 | WP_012764127.1 | WP_012764128.1 | WP_012764122.1  WP_015854294.1 | WP_012764121.1  WP_015854293.1 | WP_012764123.1  WP_015854296.1 | WP_035050229.1 |
| *Kosakonia radicincitans* DSM16656  [NZ_AKYD00000000.1](http://www.ncbi.nlm.nih.gov/nuccore/397168797) | WP_007374957.1 | WP_007374969.1 | WP_007374968.1 | WP_007374973.1  WP_007371726.1 | WP_035885754.1  WP_007371725.1 | WP_007374972.1  WP_007371728.1 | WP_007371727.1 |
| *Mangrovibacter* sp. MFB070  [NZ_JJMI00000000.1](http://www.ncbi.nlm.nih.gov/nuccore/NZ_JJMI00000000.1) | WP_036108306.1 | WP_036108278.1 | WP_036108280.1 | WP_036108266.1  WP_036109876.1 | WP_036108787.1  WP_036109879.1 | WP_036108269.1  WP_036109868.1 | WP_036109871.1 |
| *Raoultella ornithinolytica* BAL286  [JXXF01](http://www.ncbi.nlm.nih.gov/nuccore/JXXF00000000.1/) | KIZ46134.1 | KIZ46124.1 | KIZ46125.1 | KIZ46120.1  KIZ46139.1 | KIZ46119.1  KIZ46140.1 | KIZ46121.1  54291.11.peg.3462 | KIZ46138.1 |
| *Raoultella terrigena* R1Gly  [NZ_LANE00000000.1](http://www.ncbi.nlm.nih.gov/nuccore/797199371) | WP_045858170.1 | WP_045858142.1 | WP_045858145.1 | WP_045858135.1  WP_045855373.1 | WP_045858133.1  WP_044347205.1 | WP_045858136.1  WP_045855375.1 | WP_045855374.1 |
| *Thiorhodococcus drewsii* AZ1  [NZ_AFWT00000000.1](http://www.ncbi.nlm.nih.gov/nuccore/NZ_AFWT00000000.1) | WP_007042569.1 | WP_007041472.1 | WP_007041471.1 | WP_007041618.1  WP_007041959.1 | WP_007041619.1  WP_007041956.1 | WP_007041617.1  WP_007041961.1 | WP_007041960.1 |
| *Tolumonas lignolytica* BRL6-1  [NZ_AZUK00000000.1](http://www.ncbi.nlm.nih.gov/nuccore/NZ_AZUK00000000.1) | WP_051449035.1 | WP_024872646.1 | WP_024872647.1 | WP_024872642.1  WP_024873202.1 | WP_024872641.1  --- | WP_024872643.1  WP_024873204.1 | WP_024873203.1 |
|  |  |  |  |  |  |  |  |
| Verrucomicrobia |  |  |  |  |  |  |  |
| *Diplosphaera colitermitum* TAV2  [NZ_ABEA00000000.3](http://www.ncbi.nlm.nih.gov/nuccore/484112330) | WP_043581896.1 | ObacDRAFT_9080 | WP_052360782.1 | ObacDRAFT_9065  WP_043589563.1 | WP_043581909.1  WP_043589594.1 | WP_043581908.1  WP_043589561.1 | WP_043589562.1 |
| *Opitutacea bacterium* TAV5  [NZ_CP007053.1](http://www.ncbi.nlm.nih.gov/nuccore/NZ_CP007053.1) | AHF93735.1 | AHF93733.1 | --- | AHF89132.1  AHF89180.1 | AHF89131.1  AHF89179.1 | AHF89133.1  AHF89182.1 | AHF89181.1 |
|  |  |  |  |  |  |  |  |
| Euryarchaeota |  |  |  |  |  |  |  |
| *Methanosarcina siciliae* Hl350  [CP009507.1](http://www.ncbi.nlm.nih.gov/nuccore/CP009507.1) | AKB34106.1 | AKB33865.1 | AKB33866.1 | AKB33863.1  AKB33577.1 | AKB33860.1  AKB33574.1 | AKB33864.1  AKB33579.1 | AKB33578.1 |
| *Methanosarcina vacuolata* Z-761  [NZ_CP009520.1](http://www.ncbi.nlm.nih.gov/nuccore/NZ_CP009520.1) | AKB43186.1 | AKB43222.1 | AKB43221.1 | AKB43224.1  AKB44498.1 | AKB43227.1  AKB44501.1 | AKB43223.1  AKB44496.1 | AKB44497.1 |

Accession numbers from ([Dos Santos et al](#_ENREF_1)) are not listed as they can be easily accessed in the supplemental materials. GenBank accessions are listed where possible. A few genes from *Diplosphaera colitermitum* TAV2 and *Raoultella ornithinolytica* BAL286 were not deposited in GenBank, accessions are from the Pathosystems Resource Integration Center instead. ^ NifH/AnfH/VnfH designation are indicated based on best BLASTP and co-location with other alternative or canonical nitrogenase genes, but are not definitive. ^†^NifN and NifB appear to be fused genes in this organism, with a NifB domain and the N-terminus and a NifN domain at the C-terminus. We checked *A. woodii* nulcleotide sequences for possible misannotation but did not find any evidence of stop codons. Both domains were in frame and contained between a start and stop codon, suggesting a fusion. Further investigation is needed for the other organisms.* This gene has an NifE domain at the N-terminus in addition to a NifN domain at the C-terminus.
