# Supplementary material for: Diversity and Activity of Alternative Nitrogenases in Sequenced Genomes and Coastal Environments
Source: Front Microbiol. 2017 Feb 28;8:267. doi: 10.3389/fmicb.2017.00267 (PMC5328986; doi:10.3389/fmicb.2017.00267)
Supplement: Supplementary file 2 [file Table_2.docx]

**Supplementary Table S2**. Organisms with Alternative Nitrogenases.

| Species | Habitat/Isolation | Isolation | Alternative  Nitrogenase |
| --- | --- | --- | --- |
| Bacteroidetes |  |  |  |
| *Dysgonomonas capnocytophagoides* DSM 22835 | Human gall bladder | [1](#_ENREF_1) | This study |
| *Paludibacter propionicigenes* WB4 | Rice Field | [2](#_ENREF_2) | [3](#_ENREF_3) |
|  |  |  |  |
| Chlorobi |  |  |  |
| *Chloroherpeton thalassium* ATCC 35110 | Coastal Sediment (WH) | [4](#_ENREF_4) | [3](#_ENREF_3) |
|  |  |  |  |
| Cyanobacteria |  |  |  |
| *Anabaena variabilis* ATCC 29413 | Freshwater | [5](#_ENREF_5) | [3](#_ENREF_3), [6](#_ENREF_6) |
| *Peltigera aphthosa* cyanobiont ASJ3 | Lichen | [7](#_ENREF_7) | [7](#_ENREF_7) |
| *Peltigera aphthosa* cyanobiont SRDN2 | Lichen | [7](#_ENREF_7) | [7](#_ENREF_7) |
| *Peltigera aphthosa* cyanobiont S-329-1-b | Lichen | [8](#_ENREF_8) | [8](#_ENREF_8) |
| *Peltigera aphthosa* cyanobiont S-330-1-b | Lichen | [8](#_ENREF_8) | [8](#_ENREF_8) |
| *Peltigera dolichorhiza* cyanobiont | Lichen | [9](#_ENREF_9) | [9](#_ENREF_9) |
| *Peltigera membranacea* cyanobiont | Lichen | [9](#_ENREF_9) | [9](#_ENREF_9) |
|  |  |  |  |
| Firmicutes |  |  |  |
| *Acetobacterium woodii* DSM1030 | Coastal Sediment (WH) | [10](#_ENREF_10) | This Study |
| *Clostridium kluyveri* DSM555 | Sediments | [11](#_ENREF_11) | [3](#_ENREF_3), [12](#_ENREF_12) |
| *Clostridium pasteurianum* BC1 (ATCC 53464) | Coal residue | NF | This study, [13](#_ENREF_13) (other strains) |
| *Clostridium saccharoperbutylacetonicum* N1-4 (HMT) DSM14923 | Termite Hindgut | [14](#_ENREF_14) | This Study |
| *Clostridium termitidis* CT1112, DSM5398 | Termite Hindgut | [15](#_ENREF_15) | This Study |
| *Ethanoligenens harbinense* YUAN-3 | Molasses Wastewater | [16](#_ENREF_16) | [3](#_ENREF_3) |
| *Paenibacillus borealis* DSM13188 | Spruce Forest Soil | [17](#_ENREF_17) | This Study |
| *Paenibacillus camerounensis* G4 | Gorilla Stool | [18](#_ENREF_18) | This Study |
| *Paenibacillus durus* DSM1735 (*Bacillus azotofixans*, *Clostridium durum*,  *Paenibacillus azotofixans, Paenibacillus durum*)[^19-21^](#_ENREF_19) | Black Sea Sediment | [22](#_ENREF_22) | This Study |
| *Paenibacillus* sp. FSL H7-0357 | Milk | [23](#_ENREF_23) | This Study |
| *Paenibacillus sp.* HW567 |  |  | This Study |
| *Paenibacillus riograndensis* SBR5 | Rhizosphere | [24](#_ENREF_24) | [25](#_ENREF_25) |
| *Paenibacillus stellifer* DSM14472 | Paperboard | [26](#_ENREF_26) | This Study |
| *Pelosinus fermentans* R7 | Kaolin Clays | [27](#_ENREF_27) | [28](#_ENREF_28) |
| *Propionispira raffinosivoran* DSM20765 (*Zymophilus raffinosivorans*)[^29^](#_ENREF_29) | Spoiled Beer | [30](#_ENREF_30) | This Study |
| *Syntrophobotulus glycolicus* DSM8271 (FlGlyR) | Sewage Sludge | [31](#_ENREF_31) | [3](#_ENREF_3) |
|  |  |  |  |
| Proteobacteria (α) |  |  |  |
| *Azospirillum brasilense* sp 245 | Wheat Roots | [32](#_ENREF_32) | This Study, [33](#_ENREF_33)(other strains) |
| *Methylocystis parvus* OBBP | Mud/Freshwater | [34](#_ENREF_34) | This Study |
| *Pararhodospirillum photometricum* DSM122 (*Rhodospirillum photometricum*)[^35^](#_ENREF_35) | Canal | [36](#_ENREF_36) | This Study |
| *Phaeospirillum fulvum* MGU-K5 (*Rhodospirillum fulvum)*[^37^](#_ENREF_37) | Freshwater | [38](#_ENREF_38) | This Study |
|  |  |  |  |
| *Phaeospirillum molischianum* DSM120 (*Rhodospirillum molischianum*)[^37^](#_ENREF_37) | Canal | [39](#_ENREF_39) | [40](#_ENREF_40) |
| *Rhodobacter capsulatus* SB1003, ATCC BAA_309 | Soil | [39](#_ENREF_39) | [3](#_ENREF_3) |
| *Rhodomicrobium udaipurense* JA643 | Freshwater | [41](#_ENREF_41) | [41](#_ENREF_41) |
| *Rhodomicrobium vannielii* ATCC 17100 | Mud | [42](#_ENREF_42) | [3](#_ENREF_3) |
| *Rhodopseudomonas palustris* CGA009 | Canal | [38](#_ENREF_38), [39](#_ENREF_39) | [3](#_ENREF_3), [43](#_ENREF_43),44 |
| *Rhodospirillum rubrum* S1 ATCC 11170 | Canal | [39](#_ENREF_39) | [3](#_ENREF_3), [44](#_ENREF_44), [45](#_ENREF_45) |
| *Rhodovulum* sp. PH10 | Mangroves | [46](#_ENREF_46) | This Study |
|  |  |  |  |
| Proteobacteria (δ) |  |  |  |
| *Desulfobacter curvatus* DSM 3379,  AcRM3, ATCC 43919 | Marine Sediments | [47](#_ENREF_47) | [28](#_ENREF_28) |
| *Desulfobulbus elongates* DSM 2908, ATCC43118 | Digestor | [48](#_ENREF_48) | This study |
| *Desulfovibrio termitidis* Hl1 | Termite Hindgut | [49](#_ENREF_49) | This study |
| *Sulfurospirillum multivorans* DSM 12446 (*Dehalospirillum multivorans*)[^50^](#_ENREF_50) | Sewage Sludge | [51](#_ENREF_51) | [52](#_ENREF_52) |
|  |  |  |  |
| Proteobacteria (γ) |  |  |  |
| *Azotobacter chroococcum* NCIMB8003 | Soil | [53](#_ENREF_53) | [3](#_ENREF_3), [54](#_ENREF_54) |
| *Azotobacter vinelandii* DJ | Soil | [55](#_ENREF_55) | [3](#_ENREF_3), [56](#_ENREF_56), [57](#_ENREF_57) |
| *Dickeya dadantii* Ech703  (*Erwinia chrysanthemi*)[^58^](#_ENREF_58) | Plant (Pathogen) | [59](#_ENREF_59)* | [3](#_ENREF_3) |
| *Dickeya paradisiaca* NCPPB 2511 | Plant (Pathogen) | [60](#_ENREF_60) | This study |
| *Kosakonia radicincitans* DSM16656 (*Enterobacter radicincitans, Pantoea agglomerans*)[^61^](#_ENREF_61) | Plant (Endophyte) | [62](#_ENREF_62) | [28](#_ENREF_28) |
| *Magrovibacter* sp. MFB070 | Aquaculture | [63](#_ENREF_63) | This study |
| *Raoultella ornithinolytica* BAL286 | Baltic Sea | [64](#_ENREF_64) | [64](#_ENREF_64) |
| *Raoultella terrigena* R1Gly | Plant (Endophyte) | [65](#_ENREF_65) | This Study |
| *Thiorhodococcus drewsii* AZ1 | Coastal Sediment (WH) | [66](#_ENREF_66) | This Study |
| *Tolumonas lignolytica* BRL6-1 | Rainforest Soil | [67](#_ENREF_67) | This Study |
|  |  |  |  |
| Verrucomicrobia |  |  |  |
| *Diplosphaera colitermitum* TAV2 | Termite hindgut | [68](#_ENREF_68) | [69](#_ENREF_69) |
| *Opitutacea bacterium* TAV5 | Termite hindgut | [70](#_ENREF_70) | This Study |
|  |  |  |  |
| Euryarchaeota |  |  |  |
| *Methanobacterium lacus* AL-21 | Peatlands | [71](#_ENREF_71) | [3](#_ENREF_3) |
| *Methanosarcina acetivorans* C2A | Marine Sediments | [72](#_ENREF_72) | [3](#_ENREF_3), [72](#_ENREF_72) |
| *Methanosarcina barkeri* Fusaro | Freshwater Lake | [73](#_ENREF_73) | [3](#_ENREF_3) |
| *Methanosarcina siciliae* Hl350 | Oil Well | [74](#_ENREF_74) | This study |
| *Methanosarcina vacuolata* Z-761 | Digestor | [75](#_ENREF_75) | This study |
|  |  |  |  |

Where possible the original isolation paper is listed. Previous documentation of alternative nitrogenases in each organism is also indicated. Other species names used for the organism are listed in parenthesis, followed by relevant citation for renaming. NF: no isolation paper found, in this case isolation location was taken from ATCC. WH: Woods Hole. *Citation for closely related *D. dadantii* 3937.

References

1 Hofstad, T. *et al.* Dysgonomonas gen. nov. to accommodate Dysgonomonas gadei sp. nov., an organism isolated from a human gall bladder, and Dysgonomonas capnocytophagoides (formerly CDC group DF-3). *International Journal of Systematic and Evolutionary Microbiology* **50**, 2189-2195, doi:10.1099/00207713-50-6-2189 (2000).

2 Ueki, A., Akasaka, H., Suzuki, D. & Ueki, K. Paludibacter propionicigenes gen. nov., sp. nov., a novel strictly anaerobic, Gram-negative, propionate-producing bacterium isolated from plant residue in irrigated rice-field soil in Japan. *International Journal of Systematic and Evolutionary Microbiology* **56**, 39-44, doi:10.1099/ijs.0.63896-0 (2006).

3 Dos Santos, P. C., Fang, Z., Mason, S. W., Setubal, J. C. & Dixon, R. Distribution of nitrogen fixation and nitrogenase-like sequences amongst microbial genomes. *BMC Genomics* **13**, 162, doi:10.1186/1471-2164-13-162 (2012).

4 Gibson, J., PFENNIG, N. & Waterbury, J. B. Chloroherpeton thalassium gen. nov. et spec. nov., a non-filamentous, flexing and gliding green sulfur bacterium. *Archives of Microbiology* **138**, 96-101, doi:10.1007/BF00413007 (1984).

5 Tischer, R. G. Pure Culture of Anabaena flos-aquae A-37. *Nature* **205**, 419-420, doi:10.1038/205419a0 (1965).

6 Thiel, T. Characterization of genes for an alternative nitrogenase in the cyanobacterium Anabaena variabilis. *Journal of bacteriology* **175**, 6276-6286 (1993).

7 Zhang, X. *et al.* Alternative nitrogenase activity in the environment and nitrogen cycle implications. *Biogeochemistry* **127**, 189-198, doi:10.1007/s10533-016-0188-6 (2016).

8 Darnajoux, R. *et al.* Alternative Nitrogenases Contribute to Biological Nitrogen Fixation in Boreal Cyanolichens. *New Phytologist, in prep* (2016).

9 Hodkinson, B. P. *et al.* Lichen-symbiotic cyanobacteria associated with Peltigera have an alternative vanadium-dependent nitrogen fixation system. *Eur. J. Phycol.* **49**, 11-19, doi:10.1080/09670262.2013.873143 (2014).

10 Balch, W. E., Schoberth, S., Tanner, R. S. & Wolfe, R. S. Acetobacterium, a New Genus of Hydrogen-Oxidizing, Carbon Dioxide-Reducing, Anaerobic Bacteria. *International Journal of Systematic and Evolutionary Microbiology* **27**, 355-361, doi:10.1099/00207713-27-4-355 (1977).

11 Barker, H. A. The production of caproic and butyric acids by the methane fermentation of ethyl alcohol. *Archiv für Mikrobiologie* **8**, 415-421, doi:10.1007/BF00407210 (1937).

12 Seedorf, H. *et al.* The genome of Clostridium kluyveri, a strict anaerobe with unique metabolic features. *Proceedings of the National Academy of Sciences of the United States of America* **105**, 2128-2133, doi:10.1073/pnas.0711093105 (2008).

13 Zinoni, F., Robson, R. M. & Robson, R. L. Organization of potential alternative nitrogenase genes from Clostridium pasteurianum. *Biochimica et Biophysica Acta (BBA) - Gene Structure and Expression* **1174**, 83-86, doi:10.1016/0167-4781(93)90096-V (1993).

14 Poehlein, A., Krabben, P., Dürre, P. & Daniel, R. Complete Genome Sequence of the Solvent Producer Clostridium saccharoperbutylacetonicum Strain DSM 14923. *Genome Announcements* **2**, e01056-01014-e01056-01014, doi:10.1128/genomeA.01056-14 (2014).

15 Hethener, P., Brauman, A. & Garcia, J.-L. Clostridium termitidis sp. nov., a Cellulolytic Bacterium from the Gut of the Wood-feeding Termite, Nasutitermes lujae. *Systematic and Applied Microbiology* **15**, 52-58, doi:10.1016/S0723-2020(11)80138-4 (1992).

16 Xing, D. *et al.* Ethanoligenens harbinense gen. nov., sp. nov., isolated from molasses wastewater. *International Journal of Systematic and Evolutionary Microbiology* **56**, 755-760, doi:10.1099/ijs.0.63926-0 (2006).

17 Elo, S. *et al.* Paenibacillus borealis sp. nov., a nitrogen-fixing species isolated from spruce forest humus in Finland. *International Journal of Systematic and Evolutionary Microbiology* **51**, 535-545, doi:10.1099/00207713-51-2-535 (2001).

18 Keita, M. B. *et al.* Non-contiguous-Finished Genome Sequence and Description of Paenibacillus camerounensis sp nov. *Microbial Ecology* **71**, 990-998, doi:10.1007/s00248-015-0722-4 (2016).

19 Collins, M. D. *et al.* The Phylogeny of the Genus Clostridium: Proposal of Five New Genera and Eleven New Species Combinations. *International Journal of Systematic and Evolutionary Microbiology* **44**, 812-826, doi:10.1099/00207713-44-4-812 (1994).

20 Yamamoto, S., Okujo, N., Yoshida, T., Matsuura, S. & Shinoda, S. Structure and Iron Transport Activity of Vibrioferrin, a New Siderophore of Vibrio parahaemolyticus. *Journal of Biochemistry* **115**, 868-874 (1994).

21 Euzéby, J. P. Taxonomic note: necessary correction of specific and subspecific epithets according to Rules 12c and 13b of the International Code of Nomenclature of Bacteria …. *International Journal of Systematic and …* (1998).

22 Smith, L. D. & Cato, E. P. Clostridium durum, sp. nov., the predominant organism in a sediment core from the Black Sea. *Canadian Journal of Microbiology* **20**, 1393-1397, doi:10.1139/m74-214 (1974).

23 Huck, J. R., Woodcock, N. H., Ralyea, R. D. & Boor, K. J. Molecular Subtyping and characterization of psychrotolerant endospore-forming bacteria in two New York state fluid milk processing systems. *Journal of Food Protection* **70**, 2354-2364 (2007).

24 Beneduzi, A. *et al.* Paenibacillus riograndensis sp. nov., a nitrogen-fixing species isolated from the rhizosphere of Triticum aestivum. *International Journal of Systematic and Evolutionary Microbiology* **60**, 128-133, doi:10.1099/ijs.0.011973-0 (2010).

25 Fernandes, G. d. C., Trarbach, L. J., de Campos, S. B., Beneduzi, A. & Passaglia, L. M. Alternative nitrogenase and pseudogenes: unique features of the Paenibacillus riograndensis nitrogen fixation system. *Research in microbiology* **165**, 571-580 (2014).

26 Suominen, I. *et al.* Paenibacillus stellifer sp. nov., a cyclodextrin-producing species isolated from paperboard. *International Journal of Systematic and Evolutionary Microbiology* **53**, 1369-1374, doi:10.1099/ijs.0.02277-0 (2003).

27 Shelobolina, E. S. *et al.* Geobacter pickeringii sp. nov., Geobacter argillaceus sp. nov. and Pelosinus fermentans gen. nov., sp. nov., isolated from subsurface kaolin lenses. *International Journal of Systematic and Evolutionary Microbiology* **57**, 126-135, doi:10.1099/ijs.0.64221-0 (2007).

28 Glazer, A. N., Kechris, K. J. & Howard, J. B. in *Biological Nitrogen Fixation* Vol. 1 (ed Frans J de Bruijn) 87-100 (John Wiley & Sons, 2015).

29 Ueki, A., Watanabe, M., Ohtaki, Y., Kaku, N. & Ueki, K. Description of Propionispira arcuata sp. nov., isolated from a methanogenic reactor of cattle waste, reclassification of Zymophilus raffinosivorans and Zymophilus paucivorans as Propionispira raffinosivorans comb. nov. and Propionispira paucivorans comb. nov. and emended description of the genus Propionispira. *International Journal of Systematic and Evolutionary Microbiology* **64**, 3571-3577, doi:10.1099/ijs.0.063875-0 (2014).

30 Schleifer, K. H. *et al.* Taxonomic Study of Anaerobic, Gram-Negative, Rod-Shaped Bacteria from Breweries: Emended Description of Pectinatus cerevisiiphilus and Description of Pectinatus frisingensis sp. nov., Selenomonas lacticifex sp. nov., Zymophilus raffinosivorans gen. nov., sp. nov., and Zymophilus paucivorans sp. nov. *International Journal of Systematic and Evolutionary Microbiology* **40**, 19-27, doi:10.1099/00207713-40-1-19 (1990).

31 Friedrich, M., Springer, N., Ludwig, W. & Schink, B. Phylogenetic Positions of Desulfofustis glycolicus gen. nov., sp. nov. and Syntrophobotulus glycolicus gen. nov., sp. nov., Two New Strict Anaerobes Growing with Glycolic Acid. *International Journal of Systematic and Evolutionary Microbiology* **46**, 1065-1069, doi:10.1099/00207713-46-4-1065 (1996).

32 Baldani, V. L. D., Baldani, J. I. & Döbereiner, J. Effects of Azospirillum inoculation on root infection and nitrogen incorporation in wheat. *Canadian Journal of Microbiology* **29**, 924-929, doi:10.1139/m83-148 (1983).

33 Chakraborty, B. & Samaddar, K. R. Evidence for the occurrence of an alternative nitrogenase system in Azospirillum brasilense. *FEMS Microbiology Letters* **127**, 127-131, doi:10.1111/j.1574-6968.1995.tb07461.x (1995).

34 Whittenbury, R., Phillips, K. C. & Wilkinson, J. F. Enrichment, isolation and some properties of methane-utilizing bacteria. *Journal of general microbiology* **61**, 205-218, doi:10.1099/00221287-61-2-205 (1970).

35 Lakshmi, K. V. N. S., Divyasree, B., Ramprasad, E. V. V., Sasikala, C. & Ramana, C. V. Reclassification of Rhodospirillum photometricum Molisch 1907, Rhodospirillum sulfurexigens Anil Kumar et al. 2008 and Rhodospirillum oryzae Lakshmi et al. 2013 in a new genus, Pararhodospirillum gen. nov., as Pararhodospirillum photometricum comb. nov., Pararhodospirillum sulfurexigens comb. nov. and Pararhodospirillum oryzae comb. nov., respectively, and emended description of the genus Rhodospirillum. *International Journal of Systematic and Evolutionary Microbiology* **64**, 1154-1159, doi:10.1099/ijs.0.059147-0 (2014).

36 Giesberger, G. Some observations on the culture, physiology and morphology of some brown-red Rhodospirillum-species. *Antonie Van Leeuwenhoek* **13**, 135-148, doi:10.1007/BF02272755 (1947).

37 Imhoff, J. F., Petri, R. & Süling, J. Reclassification of species of the spiral-shaped phototrophic purple non-sulfur bacteria of the α-Proteobacteria: description of the new genera Phaeospirillum gen. nov., Rhodovibrio gen. nov., Rhodothalassium gen. nov. and Roseospira gen. nov. as well as transfer of Rhodospirillum fulvum to Phaeospirillum fulvum comb. nov., of Rhodospirillum molischianum to Phaeospirillum molischianum comb. nov., of Rhodospirillum salinarum to Rhodovibrio salinarum comb, nov., of Rhodospirillum sodomense to Rhodovibrio sodomensis comb. nov., of Rhodospirillum salexigens to Rhodothalassium salexigens comb. nov. and of Rhodospirillum mediosalinum to Roseospira mediosalina comb. nov. *International Journal of Systematic and Evolutionary Microbiology* **48**, 793-798, doi:10.1099/00207713-48-3-793 (1998).

38 Van Niel, C. B. The culture, general physiology, morphology, and classification of the non-sulfur purple and brown bacteria. *Bacteriological reviews* **8**, 1-118 (1944).

39 Molisch, H. *Die Purpurbakterien nach neuen Untersuchungen: eine mikrobiologische Studie*. (Fischer, 1907).

40 Duquesne, K. *et al.* Draft Genome Sequence of the Purple Photosynthetic Bacterium Phaeospirillum molischianum DSM120, a Particularly Versatile Bacterium. *Journal of Bacteriology* **194**, 3559-3560, doi:10.1128/JB.00605-12 (2012).

41 Ramana, V. V., Raj, P. S., Tushar, L., Sasikala, C. & Ramana, C. V. Rhodomicrobium udaipurense sp. nov., a psychrotolerant, phototrophic alphaproteobacterium isolated from a freshwater stream. *International Journal of Systematic and Evolutionary Microbiology* **63**, 2684-2689, doi:10.1099/ijs.0.046409-0 (2013).

42 Duchow, E. & Douglas, H. C. Rhodomicrobium vannielii, a new photoheterotrophic bacterium. *Journal of Bacteriology* **58**, 409-416 (1949).

43 Oda, Y. *et al.* Functional genomic analysis of three nitrogenase isozymes in the photosynthetic bacterium Rhodopseudomonas palustris. *Journal of Bacteriology* **187**, 7784-7794, doi:10.1128/JB.187.22.7784-7794.2005 (2005).

44 Lehman, L. J. & Roberts, G. P. Identification of an alternative nitrogenase system in Rhodospirillum rubrum. *Journal of Bacteriology* **173**, 5705-5711 (1991).

45 Gronow, S. *et al.* Complete genome sequence of Paludibacter propionicigenes type strain (WB4T). *Standards in Genomic Sciences* **4**, 36-44, doi:10.4056/sigs.1503846 (2011).

46 Khatri, I., Nupur, Korpole, S., Subramanian, S. & Pinnaka, A. K. Draft genome sequence of Rhodovulum sp. strain PH10, a phototrophic alphaproteobacterium isolated from a soil sample of mangrove of Namkhana, India. *Journal of Bacteriology* **194**, 6363-6363, doi:10.1128/JB.01695-12 (2012).

47 Widdel, F. New types of acetate-oxidizing, sulfate-reducing Desulfobacter species, D. hydrogenophilus sp. nov., D. latus sp. nov., and D. curvatus sp. nov. *Archives of Microbiology* **148**, 286-291, doi:10.1007/BF00456706 (1987).

48 Samain, E., Dubourguier, H. C. & Albagnac, G. Isolation and characterization of Desuljobulbus elongatus sp. nov. from a mesophilic industrial digester. *Systematic and Applied Microbiology* **5**, 391-401, doi:10.1016/S0723-2020(84)80040-5 (1984).

49 Trinkerl, M., Breunig, A., Schauder, R. & König, H. Desulfovibrio termitidis sp. nov., a Carbohydrate-Degrading Sulfate-Reducing Bacterium from the Hindgut of a Termite. *Systematic and Applied Microbiology* **13**, 372-377, doi:10.1016/S0723-2020(11)80235-3 (1990).

50 Luijten, M. L. G. C. *et al.* Description of Sulfurospirillum halorespirans sp. nov., an anaerobic, tetrachloroethene-respiring bacterium, and transfer of Dehalospirillum multivorans to the genus Sulfurospirillum as Sulfurospirillum multivorans comb. nov. *International Journal of Systematic and Evolutionary Microbiology* **53**, 787-793, doi:10.1099/ijs.0.02417-0 (2003).

51 Scholz-Muramatsu, H., Neumann, A., Meßmer, M., Moore, E. & Diekert, G. Isolation and characterization of Dehalospirillum multivorans gen. nov., sp. nov., a tetrachloroethene-utilizing, strictly anaerobic bacterium. *Archives of Microbiology* **163**, 48-56, doi:10.1007/BF00262203 (1995).

52 Goris, T. *et al.* Insights into organohalide respiration and the versatile catabolism of Sulfurospirillum multivorans gained from comparative genomics and physiological studies. *Environmental Microbiology* **16**, 3562-3580, doi:10.1111/1462-2920.12589 (2014).

53 Beijerinck, M. W. *Sur des microbes oligonitrophiles*. Vol. 8 190-217 (Archives néer. Science (Series 2), 1903).

54 Robson, R. L. *et al.* The alternative nitrogenase of Azotobacter chroococcum is a vanadium enzyme. (1986).

55 Lipman, J. G. *Experiments on the transformation and fixation of nitrogen by bacteria*. 217-285 (New Jersey Agricultural Experimental Station Report, 1902/1903).

56 Bishop, P. E. *et al.* Nitrogen Fixation by Azotobacter vinelandii Strains Having Deletions in Structural Genes for Nitrogenase. *Science* **232**, 92-94, doi:10.1126/science.232.4746.92 (1986).

57 Chisnell, J. R., Premakumar, R. & Bishop, P. E. Purification of a second alternative nitrogenase from a nifHDK deletion strain of Azotobacter vinelandii. *Journal of Bacteriology* **170**, 27-33 (1988).

58 Samson, R. *et al.* Transfer of Pectobacterium chrysanthemi (Burkholder et al. 1953) Brenner et al. 1973 and Brenneria paradisiaca to the genus Dickeya gen. nov as Dickeya chrysanthemi comb. nov and Dickeya paradisiaca comb. nov and delineation of four novel species, Dickeya dadantii sp nov., Dickeya dianthicola sp nov., Dickeya dieffenbachiae sp nov and Dickeya zeae sp nov. *International Journal of Systematic and Evolutionary Microbiology* **55**, 1415-1427, doi:10.1099/ijs.0.02791-0 (2005).

59 Glasner, J. D. *et al.* Genome sequence of the plant pathogenic bacterium Dickeya dadantii 3937. *Journal of Bacteriology* **193**, 2076-2077, doi:10.1128/JB.01513-10 (2011).

60 Pritchard, L. *et al.* Draft Genome Sequences of 17 Isolates of the Plant Pathogenic Bacterium Dickeya. *Genome Announcements* **1**, e00978-00913, doi:10.1128/genomeA.00978-13 (2013).

61 Brady, C., Cleenwerck, I., Venter, S., Coutinho, T. & De Vos, P. Taxonomic evaluation of the genus Enterobacter based on multilocus sequence analysis (MLSA): proposal to reclassify E. nimipressuralis and E. amnigenus into Lelliottia gen. nov. as Lelliottia nimipressuralis comb. nov. and Lelliottia amnigena comb. nov., respectively, E. gergoviae and E. pyrinus into Pluralibacter gen. nov. as Pluralibacter gergoviae comb. nov. and Pluralibacter pyrinus comb. nov., respectively, E. cowanii, E. radicincitans, E. oryzae and E. arachidis into Kosakonia gen. nov. as Kosakonia cowanii comb. nov., Kosakonia radicincitans comb. nov., Kosakonia oryzae comb. nov. and Kosakonia arachidis comb. nov., respectively, and E. turicensis, E. helveticus and E. pulveris into Cronobacter as Cronobacter zurichensis nom. nov., Cronobacter helveticus comb. nov. and Cronobacter pulveris comb. nov., respectively, and emended description of the genera Enterobacter and Cronobacter. *Systematic and Applied Microbiology* **36**, 309-319, doi:10.1016/j.syapm.2013.03.005 (2013).

62 Kämpfer, P., Ruppel, S. & Remus, R. Enterobacter radicincitans sp. nov., a plant growth promoting species of the family Enterobacteriaceae. *Systematic and Applied Microbiology* **28**, 213-221, doi:10.1016/j.syapm.2004.12.007 (2005).

63 Joseph, T. C. *et al.* First Draft Genome Sequence of a Member of the Genus Mangrovibacter, Isolated from an Aquaculture Farm in India. *Genome Announcements* **2**, e01209-01214-e01209-01214, doi:10.1128/genomeA.01209-14 (2014).

64 Bentzon-Tilia, M., Severin, I., Hansen, L. H. & Riemann, L. Genomics and Ecophysiology of Heterotrophic Nitrogen-Fixing Bacteria Isolated from Estuarine Surface Water. *mBio* **6**, e00929, doi:10.1128/mBio.00929-15 (2015).

65 Schicklberger, M., Shapiro, N., Loqué, D., Woyke, T. & Chakraborty, R. Draft Genome Sequence of Raoultella terrigena R1Gly, a Diazotrophic Endophyte. *Genome Announcements* **3**, e00607-00615, doi:10.1128/genomeA.00607-15 (2015).

66 Zaar, A., Fuchs, G., Golecki, J. R. & Overmann, J. A new purple sulfur bacterium isolated from a littoral microbial mat, Thiorhodococcus drewsii sp. nov. *Archives of microbiology* **179**, 174-183 (2003).

67 Billings, A. F. *et al.* Genome sequence and description of the anaerobic lignin-degrading bacterium Tolumonas lignolytica sp. nov. *Standards in Genomic Sciences* **10**, 1, doi:10.1186/s40793-015-0100-3 (2015).

68 Stevenson, B. S., Eichorst, S. A., Wertz, J. T., Schmidt, T. M. & Breznak, J. A. New strategies for cultivation and detection of previously uncultured microbes. *Applied and Environmental Microbiology* **70**, 4748-4755, doi:10.1128/AEM.70.8.4748-4755.2004 (2004).

69 Wertz, J. T., Kim, E., Breznak, J. A., Schmidt, T. M. & Rodrigues, J. L. M. Genomic and Physiological Characterization of the Verrucomicrobia Isolate Diplosphaera colitermitum gen. nov., sp. nov., Reveals Microaerophily and Nitrogen Fixation Genes. *Applied and Environmental Microbiology* **78**, 1544-1555, doi:10.1128/AEM.06466-11 (2012).

70 Kotak, M. *et al.* Complete Genome Sequence of the Opitutaceae Bacterium Strain TAV5, a Potential Facultative Methylotroph of the Wood-Feeding Termite Reticulitermes flavipes. *Genome Announcements* **3**, e00060-00015, doi:10.1128/genomeA.00060-15 (2015).

71 Cadillo-Quiroz, H., Bräuer, S. L., Goodson, N., Yavitt, J. B. & Zinder, S. H. Methanobacterium paludis sp. nov. and a novel strain of Methanobacterium lacus isolated from northern peatlands. *International Journal of Systematic and Evolutionary Microbiology* **64**, 1473-1480, doi:10.1099/ijs.0.059964-0 (2014).

72 Galagan, J. E. *et al.* The genome of M. acetivorans reveals extensive metabolic and physiological diversity. *Genome Research* **12**, 532-542, doi:10.1101/gr.223902 (2002).

73 Kandler, O. & Hippe, H. Lack of peptidoglycan in the cell walls of Methanosarcina barkeri. *Archives of Microbiology* **113**, 57-60, doi:10.1007/BF00428580 (1977).

74 Ni, S. S. & Boone, D. R. Isolation and characterization of a dimethyl sulfide-degrading methanogen, Methanolobus siciliae HI350, from an oil well, characterization of M. siciliae T4/MT, and emendation of M. siciliae. *International journal of systematic bacteriology* **41**, 410-416, doi:10.1099/00207713-41-3-410 (1991).

75 Zhilina, T. N. *Growth of a pure Methanosarcina culture, biotype 2, on acetate*. (MICROBIOLOGY, 1978).
