# Supplementary material for: Diversity and Activity of Alternative Nitrogenases in Sequenced Genomes and Coastal Environments
Source: Front Microbiol. 2017 Feb 28;8:267. doi: 10.3389/fmicb.2017.00267 (PMC5328986; doi:10.3389/fmicb.2017.00267)
Supplement: Supplementary file 3 [file Table_3.docx]

**Supplementary Table S3**. Metal concentrations in Sippewissett Marsh and Everglades Sampling Sites

| Site | Fe, nM or ppm | Mo, nM or ppm | V, nM or ppm |
| --- | --- | --- | --- |
| EG Overlying Water | 187.94 (105.39) | 26.42 (10.92) | 10.38 (7.84) |
| EG Porewater | 7293.22 (149.93) | 63.08 (0.025) | 15.00 (0.96) |
| SM Porewater | 1963.3 (6.1)† | 495.0(2.4)† | 46.9 (8.1)† |
| EG Leaves | 270.82 (3.31) | 1.09 (0.06) | 1.24 (0.11) |
| EG Sediment | 8389.34(955.36) | 1.49 (0.03) | 18.22 (1.69) |
| SM Sediment | 2213.30 (6.64) | 2.32 (0.05) | 7.20 (0.11) |

Metal concentrations (Fe, Mo, V) are shown as nM for overlying and porewater samples and ppm in sediments and leaves. Values in parentheses are standard deviations for duplicate measurements. † Data from ([Zhang et al 2016](#_ENREF_1)).
