# Supplementary material for: Diversity and Activity of Alternative Nitrogenases in Sequenced Genomes and Coastal Environments
Source: Front Microbiol. 2017 Feb 28;8:267. doi: 10.3389/fmicb.2017.00267 (PMC5328986; doi:10.3389/fmicb.2017.00267)
Supplement: Supplementary file 4 [file Data_Sheet_1.pdf]

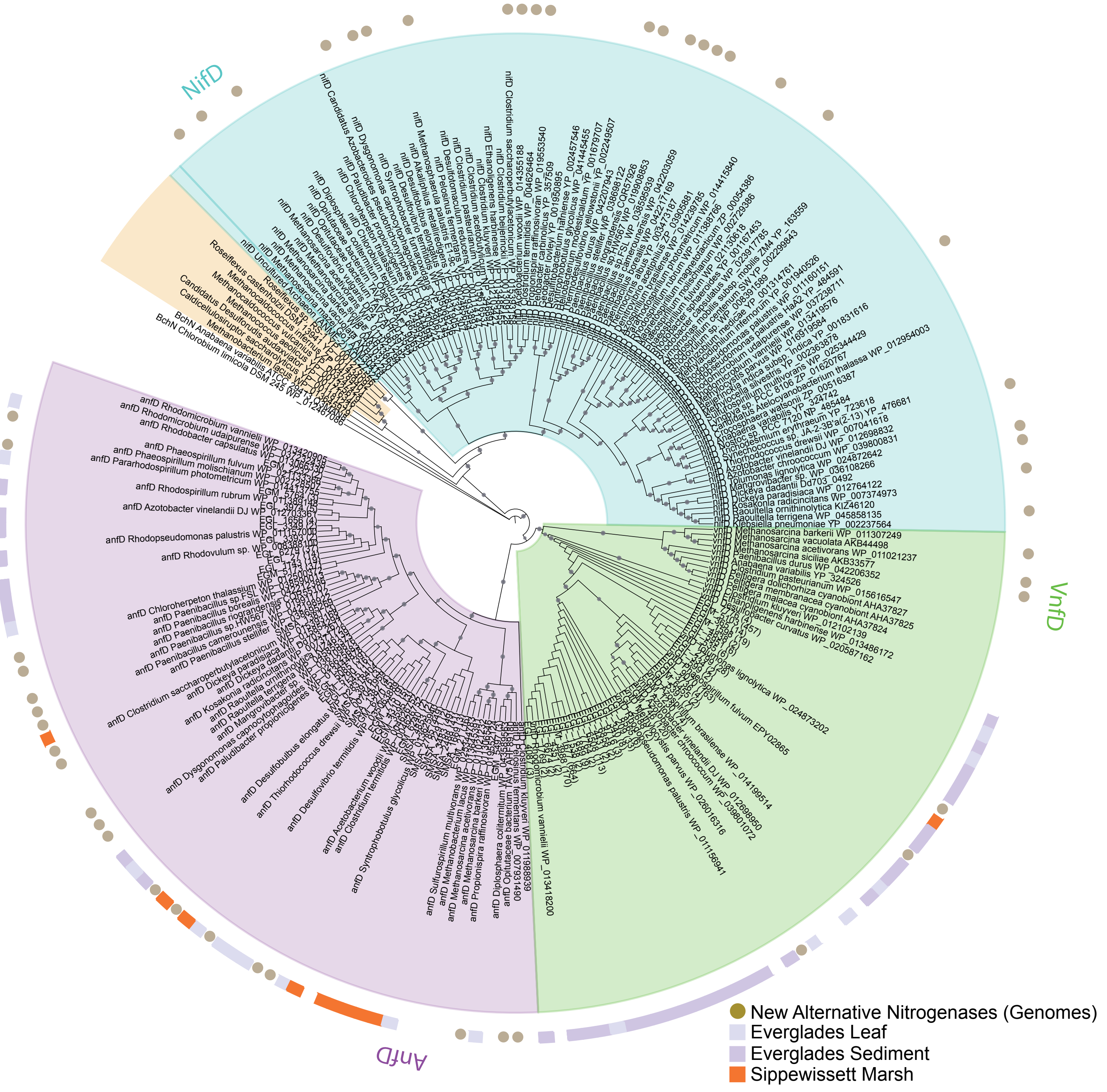

Supplementary Figure S1. Maximum likelihood phylogeny of Anf/Vnf/NifD protein sequences. Figure is the same as Figure 2, with Mo-Nitrogenases un-collapsed and without branch lengths. A reference tree was built using complete NifD, AnfD, VnfD and uncharacterized sequences. AnfD/VnfD operational taxonomic units (OTUs) recovered by this study (97% clustering, with >1 sequences per OTU) were placed using pplacer (Matsen et al 2010). The number of sequences in each OTU is shown in parentheses. ANME-2 clusters with uncharacterized nitrogenases but has been recently proposed to be a Mo-based nitrogenase (McGlynn et al 2013). Organisms with sequenced genomes for which alternative nitrogenases have been newly proposed in this study are indicated with circles in outer ring. Sampling locations for OTUs are indicated by outer ring colors: purple, Everglades leaves; light purple, Everglades sediments; orange, Sippewissett Marsh. Grey circles indicate aLRT estimates (SH > 0.6) for reference tree. EGL_2799 represents an OTU found in all environments, it was also the only V-nitrogenase OTU with >1 representative from SM and is shown in orange in the outer circle for emphasis.

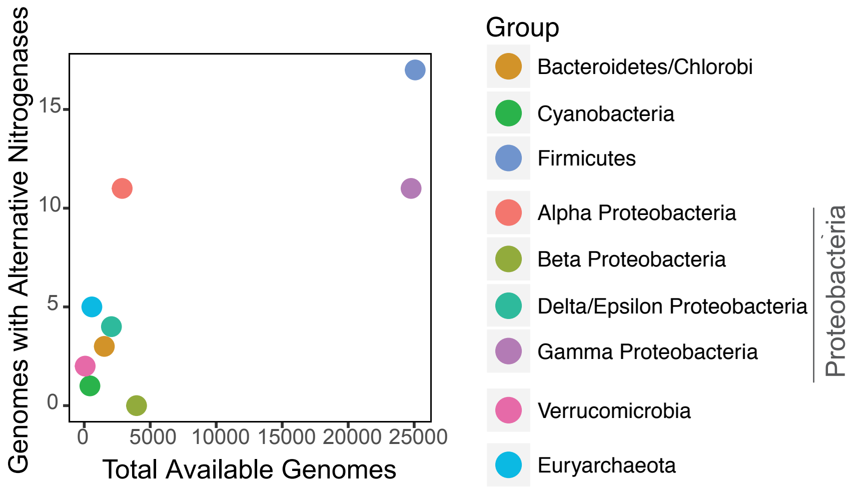

Supplementary Figure S2. Comparison of genomes with alternative nitrogenases (see Supplementary Table S2) and total available genomes in GenBank. Genome counts reflect the total number of available genomes for each group, and do not differentiate between genomes for closely related strains and those for different species.

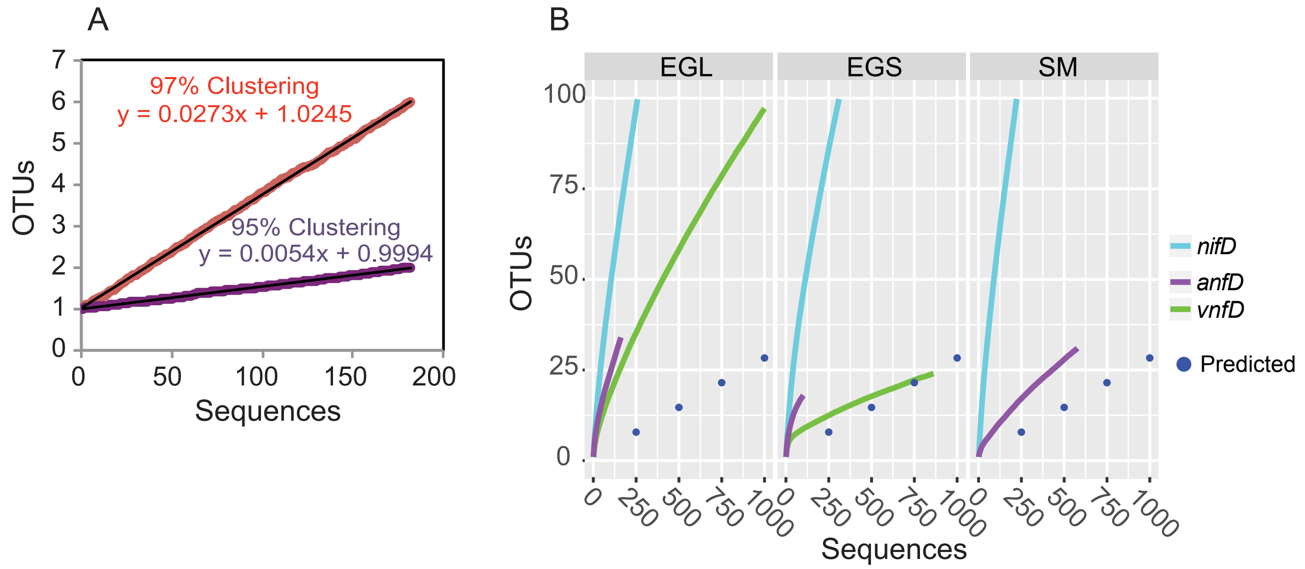

Supplementary Figure S3. Assessment of spurious OTUs due to sequencing error. (A) OTUs predicted for internal standard sequences clustered at 97% (red) and 95% (purple) and equation for line of best fit. (B) Rarefaction curves for *nifD* (blue), *vnfD* (green) and *anfD* (purple) overlain with the number of OTUs predicted from sequencing error alone (blue circles, equation from (A)). Internal standards were not screened for translation without stop codons (see methods) and likely overestimate error. EGL, Everglades leaf; EGS, Everglades sediments; SM, Sippewissett Marsh.

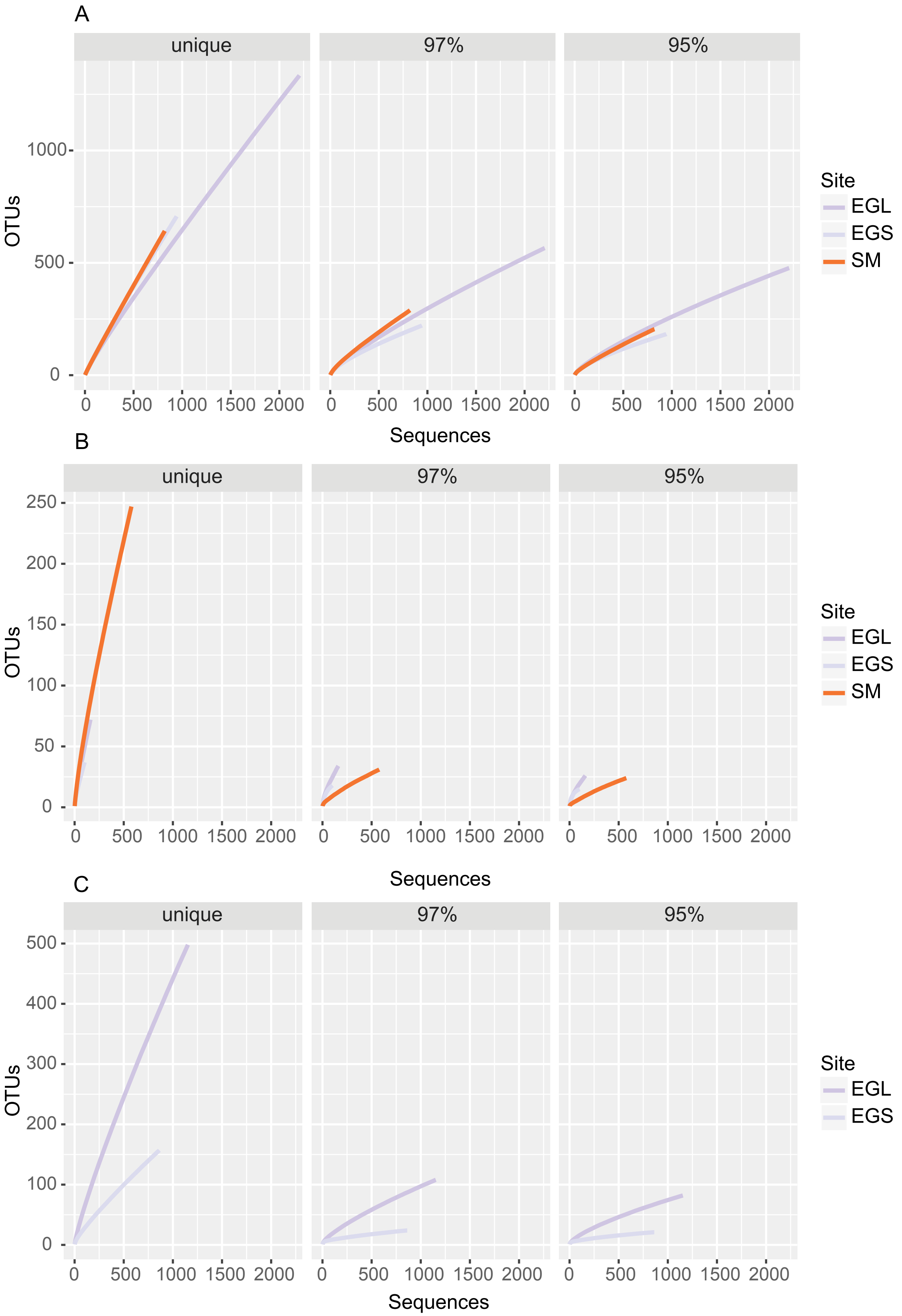

Supplementary Figure S4. Rarefaction curves for (A) *nifD*, (B) *anfD* and (C) *vnfD* at unique, 97% and 95% sequence divergence at difference sampling sites. EGL (purple), Everglades leaf; EGS (light purple), Everglades sediments; SM (orange), Sippewissett Marsh.
